# Assessment of Methods to Derive Tensile Properties of Ultra-High-Performance Fiber-Reinforced Cementitious Composites

**DOI:** 10.3390/ma17133259

**Published:** 2024-07-02

**Authors:** Tamás Mészöly, Norbert Randl

**Affiliations:** School of Civil Engineering and Architecture, Carinthia University of Applied Sciences, 9800 Spittal an der Drau, Austria; meszoely@cuas.at

**Keywords:** FRC, UHPFRC, uniaxial tensile tests, flexural tests, constitutive law

## Abstract

There is no unified method for deriving the tensile properties of fiber-reinforced ultra-high-performance cementitious composites (UHPCC). This study compares the most common material tests based on a large series of laboratory tests performed on a self-developed UHPCC mixture. The cementitious matrix, with a compressive strength of over 150 MPa and a matrix tensile strength of 8–10 MPa, was reinforced with 2% by volume of 15 mm long and 0.2 mm diameter straight high-strength steel microfibers. Over 100 uniaxial tensile tests were performed on three test configurations using cylindrical cores drilled out from larger prismatic specimens in three perpendicular directions. In addition to uniaxial tests, flexural tests on prismatic elements and flexural tests on thin plates were conducted, and the tensile properties were derived through digital image correlation (DIC) measurements and inverse analysis. Furthermore, splitting tensile tests on cylindrical specimens were employed to ascertain the tensile properties of the matrix. The outcomes of the diverse laboratory tests are presented and discussed in detail. The relationships between crack width and deflection in the context of flexural tests were developed and presented. In conjunction with compression tests and modulus of elasticity tests, the constitutive law is presented for the investigated materials.

## 1. Introduction

Fiber-reinforced concrete (FRC) exhibits advantageous properties over conventional concrete. It is able to transfer tensile stresses through cracks and to limit the crack openings by the bridging effect of the fibers in the cracks [1]. Ultra-high-performance concrete (UHPC) or ultra-high-performance cementitious composite (UHPCC) is a relatively new fine cementitious material that requires a specific combination of (at least) cement, quartz sand, quartz powder, and microsilica with a very low (around 0.2) water-to-binder ratio, and the addition of a superplasticizer [2,3]. UHPC exhibits several advantageous material properties over normal-strength concrete (NSC) or high-strength concrete (HSC). The material exhibits outstanding compressive strength, typically between 150 and 200 MPa [4,5]. Its dense material structure results in a UHPC matrix that is almost free of capillary pores, conferring the material with remarkable durability and resistance to chloride, alkali, and de-icing salt [5,6]. The high compressive strength and durability of UHPC permit the design of structures with smaller cross-sections and lower concrete cover, resulting in significant material savings compared to NSC or HSC structures. Conversely, UHPC exhibits brittle behavior under compression due to its high packing density. Consequently, UHPC is typically combined with steel fibers, resulting in a composite material known as ultra-high-performance fiber-reinforced concrete (UHPFRC). UHPFRC demonstrates ductile behavior and can resist notable tensile stresses, which can be incorporated into structural design considerations [7,8,9,10].

The use of dispersed micro-fibers allows for the effective limitation of micro-cracks in serviceability limit states (SLS) and the consideration of tensile stresses in the ultra-high-performance cementitious composite at the ultimate limit state (ULS) [5]. The use of a hybrid of fibers, a combination of different fiber materials (e.g., metallic, synthetic, mineral, or organic), sizes (e.g., micro-fibers with varying length, diameter, length-to-diameter, or micro-fibers and nano-fibers/tubes), and shapes (e.g., straight and smooth, hooked, or twisted) may also prove advantageous. It is possible to achieve improvements in material properties in different areas, such as the mechanical properties during mixing or casting of hardened concretes, durability, fire resistance, water resistance, delay of cracking, effective bridging of cracks of different sizes, and so forth [11,12,13]. Of these enhanced properties, the post-cracking behavior of the material is of particular significance. The substantial improvement in these properties has led to the emergence of a novel class of material, distinct from concrete and other stone-like materials, that exhibits considerable tensile resistance and energy absorption capacity on the tensile side, which can be incorporated into the engineering design [1]. The tensile performance can be characterized as strain-softening and strain-hardening behavior, while the flexural performance can be characterized as deflection-softening and deflection-hardening behavior. In the former case, the tensile stress–strain relationship can be used, while in the latter case, the load (or flexural stress)-deflection relationship can be used. The tensile behavior of materials can be divided into different stages as the load is increased. The first is an elastic, uncracked stage, which lasts until the first crack appears and is often defined by the point corresponding to the limit of proportionality (LOP). The second stage is the crack development stage. For materials exhibiting strain-softening behavior, this implies the presence of a limited number of cracks, potentially just a single one. In contrast, for materials characterized by strain-hardening behavior, a dense network of numerous micro-cracks is anticipated, accompanied by an increase in resistance. The third and final stage is the pull-out of fibers. It commences with crack localization, which is characterized by a gradual decline in resistance until the fibers are completely pulled out, resulting in the loss of tensile resistance [1,5,12,14,15].

However, deriving the tensile properties is not straightforward, and the process is not unified. There are various methodologies for deriving these properties, including splitting tensile tests, uniaxial tensile tests, and flexural tests. Consequently, we can discuss splitting tensile strength, uniaxial tensile strength, flexural tensile strength, and uniaxial tensile strength back-calculated from the flexural tests. Some of these methods provide only the maximum strength values, while others provide the full tensile side of the constitutive law. Furthermore, the diverse specimen shapes and dimensions required by various guidelines and standards [7,8,9,10,16,17,18] make it challenging to compare the results even from the same type of test.

Direct or indirect tensile tests can be carried out to determine the post-cracking tensile behavior. In the case of direct tensile testing (DTT) or uniaxial tensile testing (UTT), the uniaxial post-cracking tensile behavior is determined directly on tensile members. These tensile members may be unnotched or notched, rectangular or dog-bone shaped, and direct molded or extracted (e.g., cylinders drilled or prismatic specimens sawn from hardened members) [5,7,16,19,20]. The direct tensile test may be the most appropriate method to determine the tensile behavior of UHPFRC [21,22], and for UHPFRC with strain-hardening behavior, the direct tensile test is favored [5,21,23,24]. The uniaxial tensile test method recommended by RILEM for FRC [16], requires notched cylindrical specimens 150 mm in diameter and 150 mm high and is also commonly used for UHPFRC. Graybeal and Baby [25] developed a new uniaxial tensile test method for UHPFRC on prismatic test specimens that measure 17 inches (432 mm) in length and have cross-sectional dimensions of 2 inches by 2 inches (51 mm by 51 mm). This test method was recently standardized by AASHTO (American Association of State Highway and Transportation Officials) [26,27].

However, direct tensile tests are generally challenging to perform and are highly sensitive and prone to errors (specimen imperfections, boundary conditions, stress concentrations at fixation points, inhomogeneity of the material itself, etc.) [5,7,24]. The flexural test is a well-known and widely recommended testing method to determine the tensile properties of cementitious materials, especially when they are fiber-reinforced [7,8,9,23,28,29,30]. The load vs. crack mouth opening displacement (CMOD) curve or the load-bearing capacity at certain deflection can be evaluated [17]. The tests can be performed either under three-point loading [17,18,23,31] or under four-point loading (also called third-point loading) [29,32], known as a three-point bending test (TPBT) and a four-point bending test (FPBT), respectively. The specification may require the use of notched (like [17,18,23,31,33]) or unnotched (like [29,32]) specimens, or both of them (like [7,8,28]). There is a wide variety of requirements regarding the size and shape of the specimens as well: they can be square-section prismatic specimens with dimensions of 150 × 150 × 550 mm^3^ [17,18,31,32], 150 × 150 × 700 mm^3^ [29,34], 100 × 100 × 350 mm^3^ [32], 150 × 150 × 600 mm^3^, 150 × 150 × 500 mm^3^, 100 × 100 × 500 mm^3^ [33,35], 75 × 125 × 550 mm^3^ [36], 280 × 70 × 70 mm^3^, 400 × 100 × 100 mm^3^, 560 × 140 × 140 mm^3^ or 800 × 200 × 200 mm^3^ [7,8], or plate specimens with dimensions of 550 × 150 × 50 mm^3^ [10], 500 × 100 × 30 mm^3^ [9], 600 × 80 × 30 mm^3^ or 1200 × 160 × 60 mm^3^ [7,8]. The specifications also differ in some details of how the test is carried out, such as whether or not the test specimen should be rotated 90 degrees in the direction of casting.

The splitting tensile test (often referred to as the Brazilian test or split-cylinder test) is sufficient to determine the first cracking load (and so the tensile strength of the matrix), and the derived splitting tensile strength shows a good correspondence with the uniaxial tensile strength of UHPC without fibers (a conversion factor of 1.0 can be used) [6,37,38]. The test procedure may follow, for example, EN 12390-6 [39] or ASTM C496/496M [40]. In the case of UHPFRC, the load will continue to increase after cracking, and the cracks that form during cracking will be so small that identification may be challenging. Nonetheless, the splitting tensile test can also be used for UHPFRC as long as the nonlinearity point can be confidently detected. To overcome this problem, based on Nanni’s idea [41], Graybeal recommended [42] a modified test setup with measurement of the lateral deformation (expansion) of the test specimen in order to automatically identify the cracking load. The tensile strength determined in this way is important for the assessment of the SLS conditions. On the other hand, splitting tensile tests are not very useful in determining the post-cracking residual tensile strength of UHPFRC [42], which would be essential for the assessment of the ULS conditions. For splitting tensile tests of UHPFRC, cylinders with a slenderness of d/h = 2 and a minimum diameter of 100 mm should be used [6,37], and cylinders of 100 mm by 200 mm (or 4 by 8 inches in the USA) are widely adopted for these tests [42].

Beside the uniaxial tensile tests, flexural tensile tests, and splitting tensile tests, there are some other less common test methods for characterizing the tensile properties of FRC. The wedge splitting test (WST) was developed by Tschegg and Linsbauer [43,44] and adopted by Brühwiler and Wittmann [45,46]. The test has also proven to be reliable for FRC [47] and has been successfully applied to UHPC with and without fibers [48,49]. The double punch test (DPT) or double punch test method (DPTM), often referred to as the Barcelona (BCN) test, dates back to the 1970s [50,51]. It has been standardized in Spain [52], and ASTM has also developed a draft standard [53]. Recently, a simplified measurement procedure has been introduced, its applicability to UHPFRC has been validated and it has been recommended for quality control during the production of UHPFRC [54,55,56]. The double-edge wedge splitting (DEWS) test (sometimes also referred to as the double wedge splitting test or double-edge wedge test) was introduced by di Prisco et al. specifically for fiber-reinforced cementitious composites [57,58]. Recently, the test method has been successfully applied and validated to UHPFRC [59,60,61]. The round panel test (RPT) or round determinate panel (RDP) test (RDPT) (e.g., according to ASTM C1550 [62]) is also known to measure the flexural strength and energy absorption of FRC in the post-crack range [63]. Minelli and Plizzari [64,65] proposed a new test with a smaller round panel (small round determinate panel, SRDP), while Nour et al. [66] presented correction factors for thicknesses greater than the standard 75 mm. Minelli and Plizzari [67] also presented a procedure to derive a simplified stress versus crack opening law for the SRDP tests. The test has already been applied to UHPFRC in a few cases, e.g., by Lataste et al. [68]. The square panel test (SPT) or rectangular slab test was developed primarily for the measurement of the energy-absorption capacity in fiber-reinforced sprayed concrete. It can be performed in accordance with the EFNARC specification [69] and the EN 14488-5 standard [70]. Only a few applications are known for UHPFRC, e.g., Trucy et al. [71].

The objective of this study is to investigate the most commonly used experimental procedures for determining the tensile properties of ultra-high-performance fiber-reinforced composites. The results (matrix strength, residual tensile strength, dissipated energy, post-cracking curve, etc.) obtained from each test method on two UHPFRC mixtures are analyzed using statistical methods. The study examines the effect of different test setups, the influence of notching, and the effect of fiber content, fiber orientation, and fiber distribution on the measured values. Conclusions are drawn about each test method and its reliability, and the various possibilities (simplified or advanced) for defining the constitutive model from the results obtained are examined and compared. In addition, a numerical procedure for replacing crack width measurements in flexure tests is presented. The conclusions of the study can help to understand the results of the different experimental procedures and contribute to the proper definition of the constitutive model, which is essential for engineering design with ultra-high-performance fiber-reinforced composites.

In this study, the term UHPC is applied to the cementitious matrix used without fiber reinforcement, while the term UHPFRC is applied to the same matrix with fiber reinforcement.

## 2. Materials and Methods

### 2.1. UHPC

The cementitious matrix was a self-developed fine-grain UHPC [72] with a mean compressive strength derived from 100 mm cubes of more than 150 MPa without heat treatment, as measured on the 28th day after casting. The mixture had a maximum grain size of 0.4 mm. The water-to-cement ratio of the self-compacting fresh mixture was 0.23, and the water-to-binder (including 70% of the superplasticizer) ratio was 0.21. According to a parametric study published earlier [73], the density of the hardened material 28 days after casting is 2339 kg/m^3^, the mean cube compressive strength is 154.3 MPa and the mean modulus of elasticity is 44.7 GPa. Details of the test specimens, test setup, and the applied codes can be found in Section 2.8 and Section 2.9, while details of the test results can be found in Section 3.5 and Section 3.6.

### 2.2. Fiber Reinforcement

High-strength straight steel microfibers with a length of 15 mm and a diameter of 0.2 mm (a length-to-diameter ratio of 75) were employed in this study. According to the specifications provided by the manufacturer, the tensile strength of these fibers is greater than 2000 N/mm^2^.

### 2.3. UHPFRC

Combining the UHPC matrix and the chosen fiber type, two fiber-reinforced UHPC recipes were investigated in this study: one with 1 percent by volume (vol%) of fiber and one with 2 vol% of fiber. The density of the hardened material 28 days after casting is 2403 kg/m^3^ with 1 vol% of fiber and 2466 kg/m^3^ with 2 vol% of fiber. The mean compressive strength on the 28th day after casting derived from 100 mm cubes is 167.8 MPa with 1 vol% of fiber and 172.5 MPa with 2 vol% of fiber. The mean modulus of elasticity is 46.9 GPa with 1 vol% of fiber and 49.3 GPa with 2 vol% of fiber [73]. Details of the test specimens, test setup, and the applied codes can be found in Section 2.8 and Section 2.9, while details of the test results can be found in Section 3.5 and Section 3.6.

### 2.4. Splitting Tensile Tests

The splitting tensile test is an easy standard method to determine the tensile strength of cementitious materials. In the test, a cylindrical or prismatic specimen is subjected to a load perpendicular to its longitudinal axis, which induces a tensile stress perpendicular to the applied load, leading to cracking of the specimen in the plane of the loading. The test requires only a conventional compression testing machine and a steel adapter.

#### 2.4.1. Test Specimens

Cast cylindrical specimens with a diameter of 100 mm and height of 200 mm were used according to EN 12390-1 [74], produced and stored according to EN 12390-2 [75].

#### 2.4.2. Test Setup

**Figure 1:**
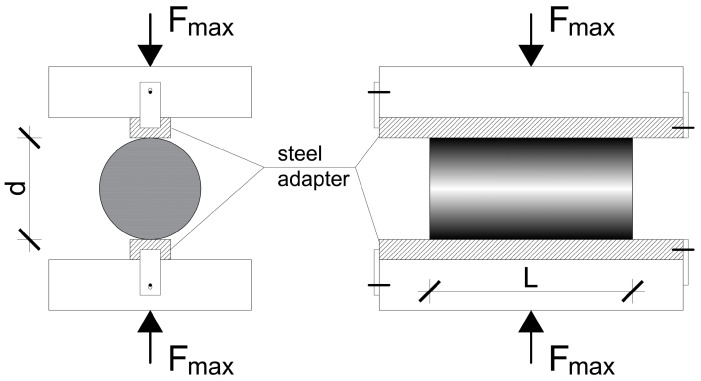
Splitting tensile test.

The test setup (see Figure 1) and test procedure were according to EN 12390-6 [39]. Unlike NSC specimens, at HSC and UHP(FR)C, no hardboard strip element is used between the test specimen and the steel adapter of the testing machine, but the steel adapter presses the cylindrical specimen directly.

The splitting tensile strength (f_ct,sp_) was calculated using the following equation:

f_ct,sp_ = 2 × F_max_/(p × L × d), (1)

where F_max_ is the maximum applied load reached during the test, L is the length of the specimen, and d is the diameter of the specimen.

### 2.5. Uniaxial Tensile Tests

A large series of uniaxial, direct tensile tests on more than one hundred cylindrical drilled cores was performed. The varying parameters were the fiber content, the drilling direction relative to the casting direction, and the test setup.

#### 2.5.1. Drilling Direction

**Figure 2:**
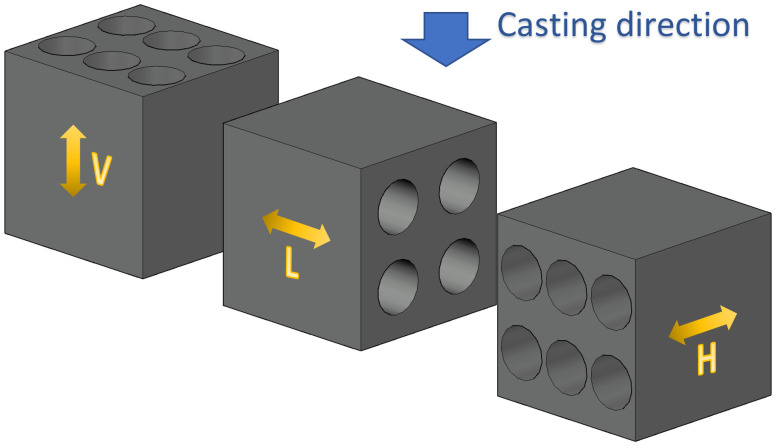
Drilling directions.

First, prismatic UHPFRC specimens were produced containing one percent and two percent of fibers by volume. The geometry of the specimens was 150 × 150 × 700 mm^3^. Second, the hardened prismatic specimens were cut into three pieces by a concrete saw. Then, the cores were drilled in horizontal (“H”; H1 to H3 in the bottom row, H4 to H6 in the top row), vertical (“V”), and longitudinal directions (“L”; with L1 to L2 in the bottom row and L3 to L4 in the top row) from the prisms. Figure 2 shows the definition of the drilling directions compared to the casting direction.

#### 2.5.2. Test Specimens

**Figure 3:**
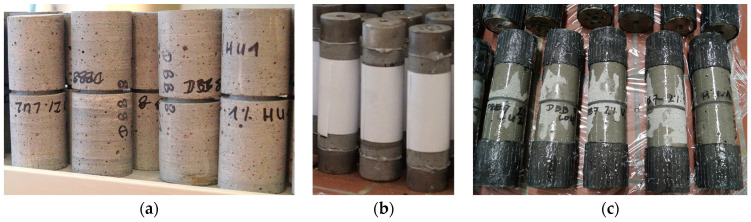
Preparation of the drilled core for testing: (**a**) Notching; (**b**) Gluing the steel adapters; (**c**) Strengthening with CFRP.

The test specimens were adapted from the RILEM guideline [16] but with a smaller diameter instead of the 150 mm specified in the RILEM guidelines to consider the dimensions of the prisms used for drilled core specimens. The nominal dimensions of the cylindrical drilled cores are 51 mm in diameter and 150 mm in length. Some specimens were tested without notching, but the majority of the specimens were notched at the middle using a concrete saw with a diamond blade and wet cutting in order to create a weakened cross-section and to concentrate the cracks and the breaking area of the specimens into that area (see Figure 3a). The width of the notch was about 3 mm and the depth of the notch was 4 mm for Series 1 and around 6.5 mm for Series 2, 3, and 4. The real dimensions were carefully measured for each specimen and applied in the calculations. The dimensions are mean values of at least three measurements.

**Figure 4:**
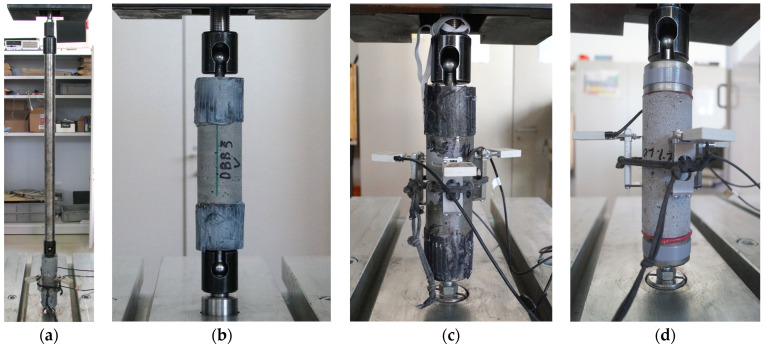
The different test setups of the uniaxial tensile tests: (**a**) Fixed and ball joints with long arm (Setup I); (**b**) Ball joints at both ends (Setup II); (**c**,**d**) Fixed and ball joints (Setup III).

Steel adapters with threaded holes at both ends of the cylindrical test specimens were attached to enable them to be fixed in the loading machine (see Figure 3b). The fixing process was as follows: cleaning the end surface of the test specimen, carefully positioning the axis of the specimen and the axis of the steel adapters into one line, and then gluing the pieces together. In order to ensure the proper connection, additional carbon-fiber-reinforced sheets (CFRP) were glued on their external surface (see Figure 3c). In the last test series, the additional strengthening could be omitted by employing a high-performance adhesive (Figure 4d).

#### 2.5.3. Test Setups

The test and measurement setup were in accordance with the recommendations of RILEM [16]. To perform the uniaxial tensile tests, considering the unavoidable imperfections, three different test setups were experienced. At the first one, a fixed pin was at one end of the specimen and a ball joint was at the other end. The end with the ball joint was connected to a 70 cm long stiff steel bar with another ball joint at the other end (see Figure 4a, later marked as Setup I). At the second test setup, ball joints were used on both sides of the specimens, directly connected to the testing machine (see Figure 4b, later marked as Setup II). At the third setup, a fixed pin on one side and a ball joint on the other side were used (see Figure 4c,d, later marked as Setup III).

The crack opening was measured using three displacement transducers with a base length of 50 mm. The three sensors were placed in the middle of the specimens in a 120-degree arrangement, and the crack opening values presented are the mean values of the three measurements.

#### 2.5.4. Testing Program

Table 1 provides a summary of the testing program, which consisted of four series of tests designed to investigate the effects of different test setups, drilling directions, and fiber content. Each series represents a separate batch of casting. Furthermore, the table indicates the number of experiments employed to ascertain the statistical values associated with each test type.

### 2.6. Flexural Tests on Prismatic Elements

In this study, the DAfStb Guideline for Steel Fiber Reinforced Concrete [29], the most widely used specification for steel-fiber-reinforced concrete structures in German-speaking countries, has been adopted.

#### 2.6.1. Test Specimens

Larger unnotched prismatic specimens were produced for the test series according to [29]. Dimensions of the test specimens were 700 × 150 × 150 mm^3^. Two test series were produced with 1 and 2 percent by volume steel fiber. The specimens for the flexural tests were cast together with other prisms used for the uniaxial tensile tests (same casting batch per fiber content).

#### 2.6.2. Test Setup

**Figure 5:**
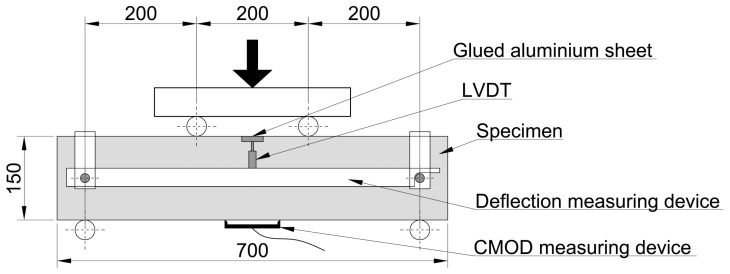
Test setup of the flexural tests on prismatic specimens [76].

A symmetric four-point test setup was used for the flexural tests, according to [29]. The distance between the supports was 600 mm, and the distance between the load introduction points was 200 mm. Figure 5 depicts the test specimen and the test setup.

### 2.7. Flexural Tests on Thin Plates

Like the flexural testing of prismatic specimens, flexural testing of plates is also a widely used method, especially in the case of applications, where the planned structural element has a plate nature, e.g., façade panels, strengthening layers, etc. This type of test was only carried out on three specimens with 2 vol% of steel fiber.

#### 2.7.1. Test Specimens

The length × width × thickness of the specimens was 700 × 150 × 30 mm^3^. They had the same dimensions as the prismatic specimens, only the height of the specimen was 30 mm instead of 150 mm.

#### 2.7.2. Test Setup

**Figure 6:**
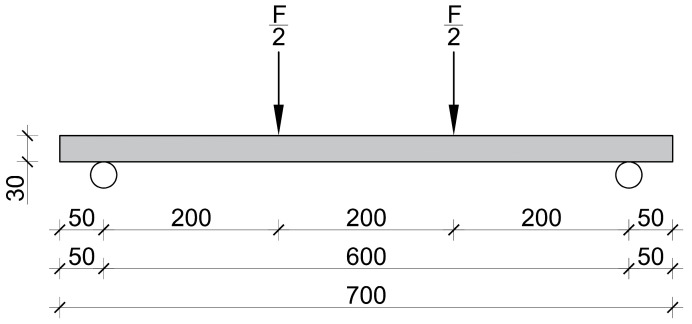
Test setup for the plate specimens.

The same test setup was used for the plate specimens as for the prismatic elements earlier; the only difference is the height of the specimens. Figure 6 shows the test specimen and the test setup used. The mid-span deflection and the applied load were measured. The bottom surface was also measured using a DIC system to derive the deformation and strain of the concrete surface and crack pattern for each load level.

### 2.8. Compression Tests

While the paper primarily focuses on the tensile properties of the investigated fiber-reinforced UHPC mixtures, in order to have a comprehensive understanding of the material’s constitutive law, it is necessary to consider the compressive side as well. To this end, compression tests on cubes were conducted.

#### 2.8.1. Test Specimens

Cube specimens with size lengths of 100 mm and cylindrical specimens with a length of 200 mm and diameter of 100 mm were produced. Sizes, production, storage, and testing were according to EN 12390-1 [74], EN 12390-2 [75], and EN 12390-3 [77], respectively.

#### 2.8.2. Test Setup

The test setup is a common compression test setup using a hydraulic jack press with a maximum capacity of 3 MN. For some of these tests, not only were the applied load values recorded, but also the specimen deformations using four laser displacement transducers. With the help of these results, the compressive strain of the specimens could be calculated based on the code recommendations.

### 2.9. Modulus of Elasticity Tests

A review of the literature reveals that the elastic modulus of UHPFRC is usually within the range of 40 to 60 GPa [9], with typical values falling between 45 and 55 GPa. [78,79] It can be assumed that the compressive and tensile side values are identical [80]. In order to obtain accurate values for the constitutive law of the investigated mixtures, the measured values of the elastic moduli in compression were examined in the laboratory.

#### 2.9.1. Test Specimens

For the tests, cylindrical specimens of 300 mm in height and 150 mm in diameter were prepared according to the specifications set forth in EN 12390-1 [74]. The production and storage of the specimens were conducted in accordance with the specifications of EN 12390-2 [75].

#### 2.9.2. Test Setup

The test setup and procedure were conducted in accordance with the specifications outlined in EN 12390-13 [81]. Strain transducers with a base length of 100 mm in a 120-degree configuration were utilized at the center of the specimen height to record the strain during the experiments.

## 3. Results

This section presents a summary of the results obtained during the experimental campaigns, with each test type considered separately.

### 3.1. Splitting Tensile Tests

A series of tests with 0 vol%, 1 vol%, and 2 vol% of fiber were performed. The mean value of the 28-day splitting tensile strength was 7.9 MPa without fiber, 14.1 MPa with 1% fiber, and 16.1 MPa with 2% fiber. For specimens containing 1% fiber, there was a clear (visual and acoustic) sign of the first cracking with a significant load drop at a splitting tensile stress of 8.1 MPa, but after that, the specimens were able to take further loading and achieved an average splitting tensile strength of 14.1 MPa. In contrast, at the 2% fiber content, there was no clearly detectable (audible or visible) failure or load drop when the matrix tensile strength was reached, and the splitting tensile strength of 16.2 MPa was reached. In this case, multiple cracking and large plastic deformations were observed. Results (with mean values, standard deviation, and coefficient of variation) are summarized in Table 2.

### 3.2. Uniaxial Tensile Tests

**Figure 7:**
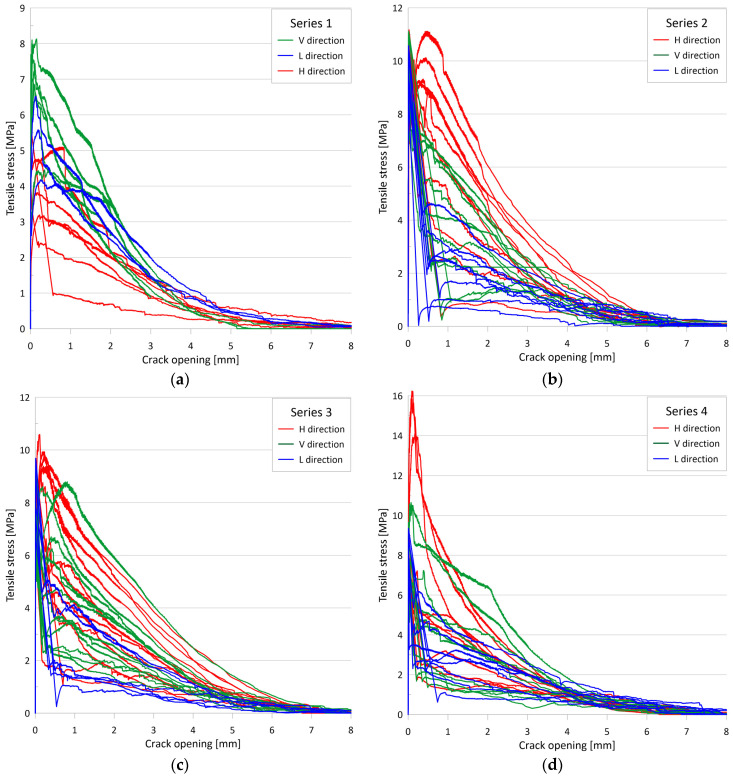
Tensile stress vs. crack opening curves for each series: (**a**) Series 1; (**b**) Series 2; (**c**) Series 3; (**d**) Series 4.

The test results represent the four series of tests performed. Setup I was used for Series 1, Setup II was used for Series 2, and Setup III was used for Series 3 and 4. Series 1, 2, and 4 included specimens containing 2% fiber by volume, while Series 3 included specimens containing 1% fiber by volume. Figure 7 shows the single tensile stress vs. crack opening for each series, with specimens in different directions marked in different colors (vertical direction in green, longitudinal direction in blue, and transverse horizontal direction in red). The graphs show that the tensile strength values have a large variance. Many specimens show a major drop in resistance after matrix cracking, while others show significant post-cracking resistance, and some even show strain-hardening behavior. The tensile resistance of specimens typically decreases to 0.2 MPa at an average crack opening of 6.1 mm and can be considered zero at an average of 7.9 mm.

Table 3 summarizes the statistical analysis of the tensile strength (f_ct_) values. The tensile strength was determined by the sudden change in the crack opening curves. In many cases, the determination is obvious; in other cases, it requires a relative analysis of the three individual crack opening curves. From the analysis, the worst results were obtained for Setup I (range 2.0–8.1 MPa, mean 5.0 MPa, median 5.1 MPa, and standard deviation 1.9 MPa), with significantly better results for Setup III (range 6.1–10.6 MPa, mean 7.9 MPa, median 7.8 MPa, and standard deviation 1.0 MPa for Series 3, while range 6.1–9.6 MPa, mean 7.9 MPa, median 7.7 MPa, and standard deviation 1.0 MPa for Series 4), and the most favorable results were obtained with Setup II (range 7.5–11.2 MPa, mean 9.8 MPa, median 9.8 MPa, and standard deviation 0.8 MPa).

Table 4 presents a summary of the statistical analysis of the post-cracking residual tensile strength (f_cf_) values. In specimens exhibiting hardening behavior after cracking, the residual strength was determined by the highest stress value in the post-cracking range. Conversely, in specimens that exhibited softening behavior, the residual tensile strength was taken to be the tensile stress corresponding to an average crack width of 0.3 mm, in accordance with [7,80]. From the analysis, the mean crack opening at residual tensile strength was 0.27 mm for Series 1, 0.75 mm for Series 2, 0.46 mm for Series 3, and 0.41 mm for Series 4, while the median crack opening at residual tensile strength was 0.20 mm for Series 1, 0.53 mm for Series 2, 0.37 mm for Series 3, and 0.30 mm for Series 4. For all tested specimens, the mean crack opening at residual tensile strength was 0.50 mm and the median crack opening was 0.37 mm. The mean residual tensile strengths for the different series were similar: 5.1 MPa, 5.0 MPa, 5.2 MPa, and 5.5 MPa for Series 1, 2, 3, and 4, respectively. The median residual tensile strength values were 5.1 MPa, 4.6 MPa, 5.0 MPa, and 4.1 MPa, while the standard deviation of the residual tensile strength was 2.0 MPa, 3.2 MPa, 2.7 MPa, and 4.2 MPa for Series 1, 2, 3, and 4, respectively.

Table 5 and Table 6 summarize the statistical analysis of the dissipated energy (W_f_) values. Dissipated energy is defined as the area under the tensile stress–crack opening curve up to a given crack width (definite integral of the tensile stress with respect to the crack opening from zero to a given crack width) [17,78]. For Table 5, this crack width was 2 mm, while for Table 6, it was 3 mm, and the resulting values are W_f,2_ and W_f,3_, respectively. The mean values of the energy dissipated up to 2 mm were 7.4 kJ/m^2^, 9.2 kJ/m^2^, 8.3 kJ/m^2^, and 8.0 kJ/m^2^, the median values of the energy dissipated up to 2 mm were 7.7 kJ/m^2^, 7.8 kJ/m^2^, 7.9 kJ/m^2^, and 9.4 kJ/m^2^, while the standard deviation of the energy dissipated up to 2 mm was 2.2 kJ/m^2^, 3.8 kJ/m^2^, 3.4 kJ/m^2^, and 3.8 kJ/m^2^ for Series 1, 2, 3 and 4, respectively.

The mean values of the energy dissipated up to 3 mm were 8.9 kJ/m^2^, 11.5 kJ/m^2^, 10.6 kJ/m^2^, and 10.1 kJ/m^2^, the median values of the energy dissipated up to 3 mm were 9.8 kJ/m^2^, 9.7 kJ/m^2^, 10.3 kJ/m^2^, and 9.7 kJ/m^2^, while the standard deviation of the energy dissipated up to 3 mm was 2.6 kJ/m^2^, 4.9 kJ/m^2^, 4.6 kJ/m^2^, and 4.7 kJ/m^2^ for Series 1, 2, 3, and 4, respectively.

### 3.3. Flexural Tests on Prismatic Elements

The primary test results of the flexural tests are the applied load vs. deflection (or the flexural tensile stress vs. crack opening) curves, the cracking load (or the matrix tensile strength), and post-cracking flexural tensile strength. Secondary results can be the uniaxial tensile stress vs. crack opening and the uniaxial tensile stress vs. tensile strain curves derived from the uniaxial tensile stress vs. crack opening (or deflection in some simplified methods) using inverse analysis (i.e., sometimes called back-analysis or inverse method). The procedure is based on the equilibrium of the cracked cross-section in bending and determines the uniaxial tensile stress for each crack width using a stepwise iterative solution. It is described in [82,83], applied by [7,8], and developed further [84]. These curves provide useful and valuable data about the real tensile properties of the investigated material, especially about the post-cracking and post-peak behavior.

**Figure 8:**
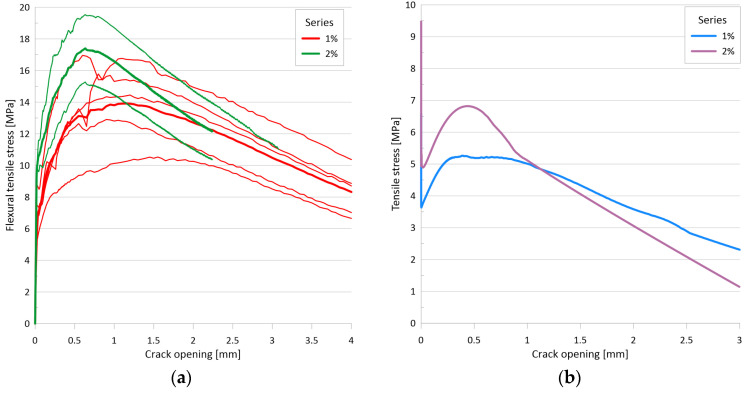
Curves derived from flexural tests on prisms: (**a**) Flexural stress-crack opening curves; (**b**) Tensile stress–crack opening relations.

Figure 8a illustrates the flexural tensile stress versus crack opening curves for the two mixtures, while Figure 8b depicts the uniaxial tensile stress versus crack opening curves for the two mixtures with 1 vol% and with 2 vol% fiber. The minimum value of the flexural tensile strength for the test series with 1% steel fiber was 10.5 MPa, while the maximum value was 17.0 MPa. The mean curve yielded a flexural tensile strength of 13.9 MPa. For a mixture with 2% steel fiber, the minimum value of the flexural tensile strength was 15.3 MPa, while the maximum value was 19.5 MPa. For the mean curve, the flexural tensile strength was 17.4 MPa. The flexural tensile strength of the mixture with 1% steel fiber was found to be associated with an average crack width of 1.15 mm, while the mixture with 2% steel fiber exhibited an average crack width of 0.63 mm at the flexural tensile strength. The tensile strength of the matrix, determined as the value of the flexural tensile stress at the first crack, was 6.6 MPa for the 1% fiber series and 9.5 MPa for the 2% fiber series. The (uniaxial) tensile stresses determined by inverse analysis exhibited a sharp decline immediately after cracking, followed by a gradual increase as the fibers became activated (see Figure 8b). The lowest stress value shortly after cracking was 3.6 MPa with 1% fiber and 4.9 MPa with 2% fiber. The local maximum of the curve after cracking was 5.3 MPa with 1% fiber and 6.8 MPa with 2% fiber. For both series, this maximum was reached at a crack width of approximately 0.4 mm.

**Figure 9:**
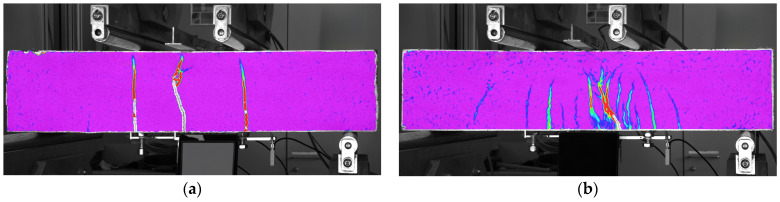
Crack patterns from DIC measurement: (**a**) with 1 vol% fiber; (**b**) with 2 vol% fiber (the colors represent the strain values; with warmer colors indicating a higher value).

Figure 9 illustrates the difference between the crack patterns at peak load for mixtures with 1% by volume (Figure 9a) and 2% by volume fiber content (Figure 9b). It can be clearly observed that at lower fiber content, fewer cracks are formed, while at higher fiber content, a dense network of microcracks is formed. This phenomenon can be attributed to the higher fiber content, which increases the likelihood of fibers being present at the crack plane. These fibers resist the applied load by bridging the micro-cracks and transferring the load to the uncracked cementitious matrix, allowing the formation of multiple micro-cracks [85]. This behavior was investigated in detail using a digital image correlation (DIC) system in [76]. It is important to note that when four-point loading was applied to the specimens without a notch as specified, some cracks were regularly found to fall outside the measured section, and several times, the decisive crack that developed after the peak load was reached also fell outside the measured section.

### 3.4. Flexural Tests on Thin Plates

**Figure 10:**
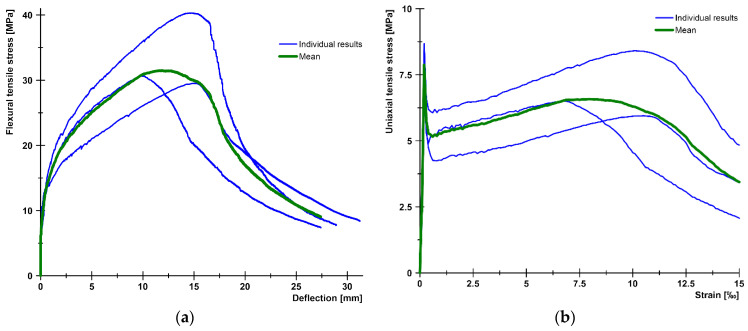
Curves derived from flexural tests on thin plates: (**a**) Flexural stress-deflection curves; (**b**) Tensile stress–strain relations.

The primary test results are the applied load vs. deflection curves and the flexural tensile stress vs. deflection curves derived from the former. Figure 10a presents the flexural tensile stress vs. deflection curves and the mean curve calculated from them. The individual maximum values are 40.3 MPa, 30.6 MPa, and 29.5 MPa, while the maximum value of the mean curve is 31.5 MPa. Based on the analysis of the DIC measurements, the flexural tensile stresses at the first crack of the plates were 14.5 MPa, 12.7 MPa, and 11.2 MPa, with an average value of 12.8 MPa. Figure 10b shows the uniaxial tensile stress–tensile strain relation derived from the flexural stress–tensile strain curves using inverse analysis. From these curves, the peak values of the elastic phase, which can be considered as the (uniaxial) tensile strength of the cementitious matrix, were 8.7 MPa, 8.5 MPa, and 6.5 MPa with a mean value of 7.9 MPa, while the maximum values of the post-cracking phase were 8.4 MPa, 6.5 MPa, and 6.0 MPa, and the maximum value corresponding to the mean curve was 6.6 MPa.

**Figure 11:**
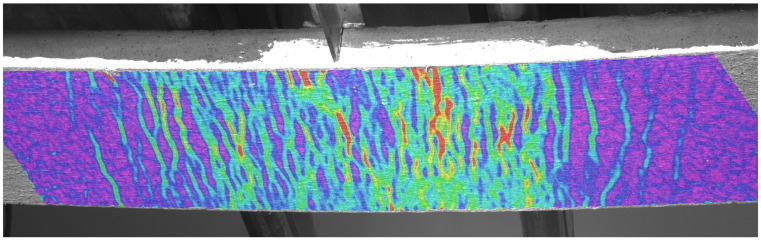
Typical crack pattern from DIC measurements (the colors represent the strain values; with warmer colors indicating a higher value).

Figure 11 illustrates the typical crack pattern of a plate specimen under flexural loading. For more information on typical crack patterns of bent UHPC plates with different reinforcement types, see [86]. As can be seen from Figure 11, steel-fiber-reinforced plates are usually characterized by a dense network of cracks, so that the crack mouth opening displacement (CMOD) measurement, which is common for prismatic specimens, cannot be used. Therefore, in this case, the strain is measured and the inverse analysis is based directly on the applied load-strain curves as described in [7,8] and resulted in the uniaxial tensile stress–strain curves as shown in Figure 10b.

### 3.5. Compressive Strength

Table 7 summarizes the results of the cube tests at an age of 28 days. More details can be found in [73] with a comparison of the cube and cylinder strength values, strength values at different ages, and a wider statistical and correlational analysis of several mechanical properties of the mixtures. The characteristic strength values determined using the Student’s coefficient (according to [8]) were 148.1 MPa without fiber reinforcement, 158.2 MPa with 1% steel fiber, and 158.9 MPa with 2% steel fiber.

**Figure 12:**
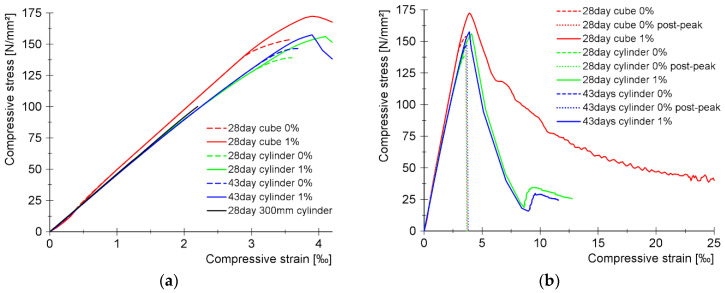
Compressive stress–strain relation [73]: (**a**) Pre-peak compressive behavior; (**b**) Post-peak compressive behavior.

Figure 12a,b show the compressive side of the constitutive law with 1 vol% fiber and without fiber for different specimen shapes and sizes. Figure 12a shows the pre-peak part of the stress–strain curves and Figure 12b shows the entire curves (focusing on the post-peak behavior). Since UHPC mixtures without fiber reinforcement tend to fail explosively, it is usually not possible to measure their post-peak behavior, and Figure 12a,b show these curves only up to the peak value.

Unfortunately, this test series did not include compression tests with deformation measurement for specimens containing 2% by volume of fiber. Therefore, a further series of specimens containing 1% and 2% by volume of steel fiber were produced and tested with deformation measurement included to compare their post-peak behavior in compression. Their compressive strength values measured on cubes at 28 days of age are summarized in Table 8.

**Figure 13:**
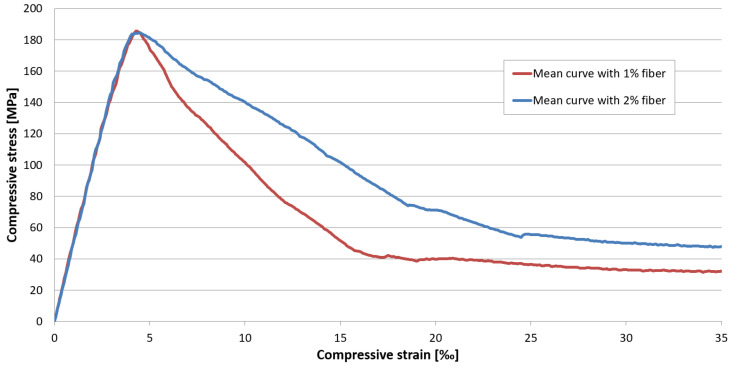
Compressive stress–strain relations.

Figure 13 presents the compressive stress–strain relations for the two mixtures. While the curves of the mixtures with different fiber content show minimal difference up to the peak value, a significant difference is seen in their post-peak range.

While the curves for non-reinforced UHPC peak at an average compressive strain of 3.5‰ (3.4–3.6‰), the curves for UHPC reinforced with 1% by volume steel fiber reach an average strain value of 3.9‰ (3.7–4.2‰) and the curves for the 2% by volume steel-fiber-reinforced UHPC average 4.1‰ (3.9–4.4‰).

### 3.6. Modulus of Elasticity

In order to determine the moduli of elasticity, three specimens were measured on 300 mm × 150 mm cylinders at an age of 28 days for each mixture. The mean values were 44.7 GPa without fiber reinforcement, 46.9 GPa with 1% by volume of steel fiber, and 49.3 GPa with 2% by volume of steel fiber. The standard deviation was found to be between 0.3 and 0.4 GPa in all three cases. The results measured on the same mixture but on specimens of different shapes, sizes, and ages are presented in [73].

Furthermore, the stress–strain curves of the compression tests performed with deformation measurements indicate a modulus of elasticity of approximately 48.0 GPa.

## 4. Discussion

This section is devoted to a comprehensive evaluation and discussion of the observed results. The discussion will proceed in two phases. Initially, the results will be presented separately for each type of test. Thereafter, a comparison will be conducted to ascertain whether the observed outcomes exhibit any patterns or consistencies and what new learnings and conclusions can be drawn.

### 4.1. Discussion on the Splitting Tensile Tests

The specimens with 1% fiber showed a clear sign of the first crack at a splitting tensile stress of 8.1 N/mm^2^. These results represent the splitting tensile strength of the matrix and show good agreement with the results obtained from the specimens without fibers, where the splitting tensile strength was 7.9 N/mm^2^. In the case of the specimens with 2 vol% of fiber, there was no significant load drop, which means that the higher amount of fibers could prevent the opening of the initial cracks.

The tests showed that 1 vol% fiber increased the splitting tensile strength by 75–80% over the same values for specimens without fiber, and 2 vol% fiber increased the splitting tensile strength by 100–105% over the same values for specimens without fiber. The additional 1 vol% fiber increased the splitting tensile strength by approximately 15% compared to specimens containing 1 vol% fiber. This is consistent with previous observations that fiber addition has a large effect on tensile properties, but this effect is not proportional to the amount of fiber added to the mixture [87].

While the value of the standard deviation remained below 1.0 MPa for the mixture without fibers, it was about 1.0 MPa for the mixture with 1 vol% fiber and about 1.2 MPa for the mixture with 2 vol% fiber. However, the coefficient of variation was about 12% for the mixture without fibers and 7.3–7.4% for the mixtures with fibers, which are relatively lower values.

### 4.2. Discussion on the Uniaxial Tensile Tests

The section presents a discussion of a number of topics based on the results of the uniaxial tensile tests performed.

#### 4.2.1. Matrix Tensile Strength

**Figure 14:**
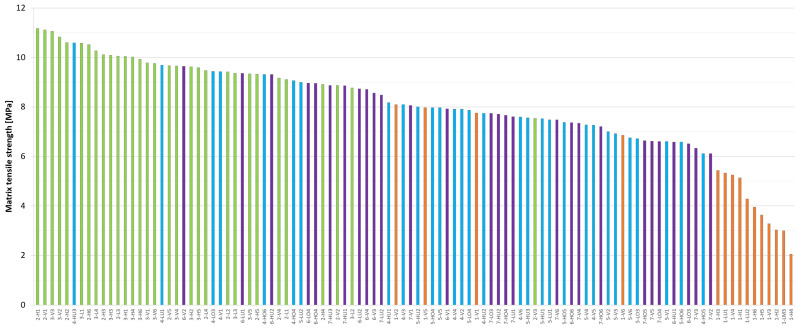
Matrix tensile strength (f_ct_) values from the uniaxial tensile tests, ordered by their value (orange: Series 1, green: Series 2, light blue: Series 3, purple: Series 4).

Figure 14 depicts the individual matrix tensile strength values of the uniaxial tests, arranged in order of value and separated by color according to the test series. A statistical summary of these values is provided in Table 3. It is evident that the matrix tensile strength values are significantly lower (5.0 MPa on average) and less reliable for the first series, with the highest values provided by the second series (9.8 MPa on average), while the third and fourth series provided intermediate values (7.9 MPa). The mean value of Series 2 is nearly twice as large (+96%) in comparison to Series 1. However, the difference in standard deviation is even more pronounced, with Series 1 exhibiting a value that is almost two and a half times greater (+144%) than that of Series 2. The results of this experiment demonstrate the significance of the test setup in uniaxial tensile tests. Furthermore, they illustrate that the setup utilized for Series 1 is inadequate for the determination of matrix strength. When comparing the values of Series 3 and 4, although the former gives the highest values and the latter the lowest, there is practically no difference between them when considering the statistical evaluation. Both the mean and the standard deviation values can be considered to be identical for a sample of more than sixty uniaxial tensile tests from these two series. This confirms the hypothesis that the matrix strength is, in fact, not affected by the amount of steel fiber used. A comparison of the second, third, and fourth series reveals that the second series exhibits a more favorable performance in terms of both measured values and their standard deviation. The mean value for Series 2 is approximately 25% higher, while the standard deviation is 25% lower. This indicates that the actual tensile strength of the mixture utilized is likely to approach 10 MPa, while Series 3 and 4 provide a mean value of 7.9 MPa with a standard deviation of 1 MPa.

The following relationship provides a conversion between the mean tensile strength of the matrix as determined by the splitting tensile tests (f_ctm,sp_) and the uniaxial tensile strength (f_ctm_) as determined by the uniaxial tensile tests using the conversion factor α_sp_:

f_ctm_ = α_sp_ × f_ctm,sp_. (2)

A comparison of the results obtained from the uniaxial tests with a matrix tensile strength value of 7.9 MPa determined from splitting tensile tests reveals that the conversion factor α_sp_ was close to 1.0 for Series 3 and 4, while it was approximately 1.2 for Series 2. These results indicate that the conversion factor between uniaxial tensile strength and splitting tensile strength should be taken as at least α_sp_ = 1.0 for UHP(FR)C, with α_sp_ = 1.2 providing the most probable tensile strength value for the given mixtures.

The displacement transducer used to determine the crack opening in the post-cracking state measured the elastic deformation in the pre-cracking state, allowing the elastic strain associated with the first crack to be determined. The measurements show that the first cracks are associated with a deformation between 8 and 10 μm, corresponding to an elongation of 0.16–0.20‰ at a base length of 50 mm. This is consistent with previous findings, as the strain values associated with Young’s modulus of 47–49 GPa and tensile strength of 8–10 MPa are also in this range. In most cases, at the moment of cracking, the crack opening suddenly jumped from 8–10 μm to a multiple of 0.1 mm while the steel fibers were activated.

#### 4.2.2. Residual Tensile Strength and Dissipated Energy

**Figure 15:**
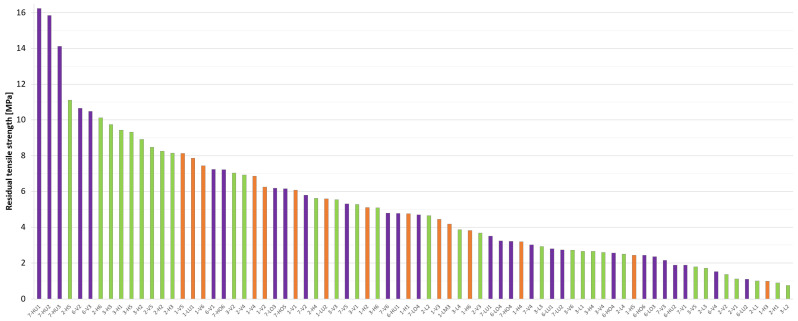
Residual tensile strength (f_cf_) values from the uniaxial tensile tests, ordered by their value (orange: Series 1/Setup I, green: Series 2/Setup II, purple: Series 4/Setup III).

Figure 15 depicts the individual residual tensile strength values of the uniaxial tests for Series 1, 2, and 4, arranged in order of value and separated by color according to the test series. As only the series with 2 vol% steel fiber are included in Figure 15, it is mainly a comparison of the three test setups. A statistical summary of these values is provided in Table 4. The initial observation is that the three values from the fourth series are significantly higher than the rest of the data. It can also be observed that there is a large difference between the extreme values of the test series. The highest value exceeds 16 MPa, while the lowest values just reach 1 MPa. Furthermore, the very low values are mixed with specimens from all three test setups.

The statistical analysis confirms that the post-cracking behavior is less affected by the test setup employed than the matrix strength. Although Series 4 appears to be the most favorable based on the mean value when the three outliers are excluded from the calculation, the mean value is only 4.3 MPa, the median value is 3.2 MPa, and the standard deviation is 2.5 MPa, making it the least favorable of the three test setups in term of residual tensile strength. Comparing the first and second test setups, the mean residual tensile strength values were 5.1 MPa for the first test setup and 5.0 MPa for the second, the median values were 5.1 MPa and 4.6 MPa, and the standard deviations were 2.0 MPa and 3.2 MPa, respectively. This implies that the first test setup, which was found to be unsuitable for measuring matrix strength, exhibited superior performance with consistent results compared to the other two test setups when investigating the post-cracking residual tensile strength. Similarly, when examining Figure 15, it appears that the values measured with the first test setup lie typically in the middle of the range.

Since the evaluation of the residual strength values suggests only moderate differences between the results obtained from the distinct test setups, the large differences observed in the reported data are expected to be due to the orientation and distribution of the steel fiber reinforcement, which provides the post-crack strength. This will be investigated in a subsequent section.

**Figure 16:**
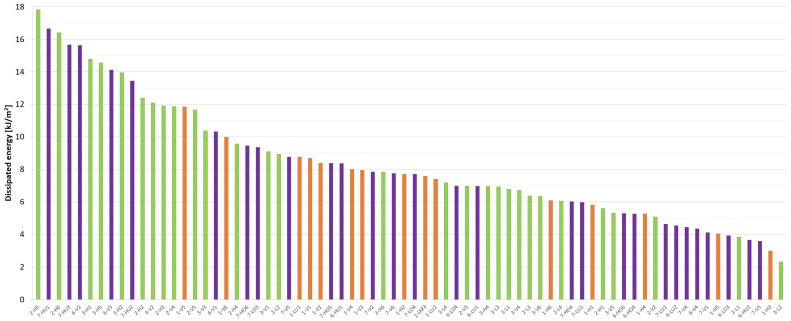
Dissipated energy (W_f,2_) values from the uniaxial tensile tests, ordered by their value (orange: Series 1/Setup I, green: Series 2/Setup II, purple: Series 4/Setup III).

Figure 16 depicts the individual dissipated energy values (W_f,2_) of the uniaxial tests up to 2 mm of crack opening, arranged in order of value and separated by color according to the test series. A comparison with Figure 15 reveals that the data for the three series (and test setups) are more evenly distributed. Furthermore, the three outlying values in terms of residual tensile strength are no longer outlying in terms of dissipated energy. This indicates that a high peak of strength value is not sufficient for this outcome; it is also necessary to ensure this within a larger range.

A statistical summary presented in Table 5 indicates that the outcomes of the first and fourth series are comparable. However, a more detailed analysis reveals that, although the fourth series appears to be more favorable in terms of the mean, it is comparatively weaker in terms of the median and standard deviation. Moreover, the mean is also slightly lower (7.10 kJ/m^2^ vs. 7.36 kJ/m^2^) in the case of the evaluation without the three outlying specimens. A comparison of the three test series reveals that Series 2 exhibits the highest values for dissipated energy, although it is less favorable in terms of standard deviation. Consequently, in contrast to the evaluation based on residual tensile strength, where Series 1 demonstrated the most favorable performance, Series 2 appears to be more favorable in terms of dissipated energy.

The energy values corresponding to a crack width of 3 mm (W_f,3_) would exhibit a comparable pattern at 25–30% higher values, with some adjacent specimens switching places. Nevertheless, the overall conclusions would remain consistent.

#### 4.2.3. Effect of the Test Setup

The efficacy of various test setups was examined based on the observation that specimens with fixed boundary conditions, as recommended in the literature [78,88,89], frequently exhibited cracking during mounting in the testing equipment or at the very beginning of the test due to constraints resulting from imperfections. While the preparation of the test specimens and the test setup was conducted with the utmost precision and care, it is inevitable that a minimal eccentricity and inclination will occur relative to the ideal axis line. However, the rigid loading equipment does not permit any geometrical deviation, which may result in significant internal forces due to these minimal imperfections. In order to reduce the impact of these initial imperfections on the test specimen, the test setups shown in Figure 4 (Section 2.5.3) were subjected to examination.

**Figure 17:**
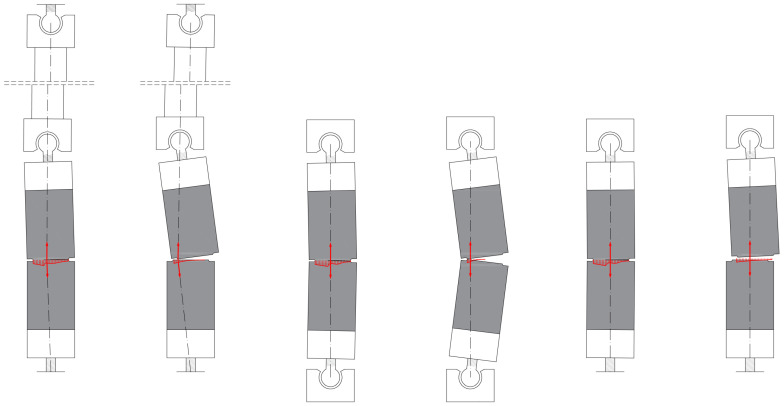
Fracture mechanism of the test specimens in case of the different test setups.

In addition to geometric imperfections, material inhomogeneity and anisotropy also play a significant role during loading in both the fracture location and the fracture process (see in Section 4.2.4). This is to a lesser extent a result of matrix inhomogeneity, but mainly a result of the distribution and orientation of the steel fibers. Therefore, even in the case of perfect geometry, the load would be eccentric, and the stress distribution non-uniform. Furthermore, additional eccentricity is induced after cracking, as it always occurs first at one edge of the specimen. Consequently, the center of the cracked cross-section is no longer aligned with the axis of the applied load (assuming a centric loading a priori). The resulting moment acts to rotate the two parts of the specimen facing the crack relative to each other and to open the crack further. If the boundary conditions allow, the test specimen parts will rotate and displace perpendicular to the load axis. This is a self-reinforcing fracture mechanism that leads to further rotation and a further increase in eccentricity during the loading process. If the boundary condition does not allow free rotation at one end of the test specimen but does at the other end, then opposing moments are induced. The bending moment due to the eccentricity from the cracking acts in the direction of the opening of the fracture surface, while the moment due to the combination of the rotation at the hinge and the tensile force acts in the direction of its closure. The combined effect of these forces results in limited relative rotation of the two parts of the test specimen, while simultaneously creating constraints (and a later stage relative displacements) parallel to the fracture surface (perpendicular to the tensile force). This kinematic response was particularly well observed in Setup I. In Setup II, the test specimens were fixed directly to the testing machine on both ends using ball joints, which prevented the end of the specimen from moving out of the vertical line and only allowed rotation around the hinges. In the third approach, designated Setup III, a fixed connection was established at one end of the specimens. This prevented not only the ends of the specimens from deviating from the vertical axis but also limited the horizontal movement of the fracture section. In addition to these kinematic responses, a further factor is introduced depending on the fiber distribution. For each loading level, the stress distribution at the fracture surface results in an equilibrium state where the line of action of the resultant force of the stress body passes through the center of the ball joints. Figure 17 illustrates this phenomenon for all three test setups, for an early cracked condition and toward the end of the load test.

If a significant proportion of the fibers show a clear orientation within the fracture surface, following the complete development of the fracture, the fibers crossing the fracture surface in a direction not parallel to the tensile force will tend to align themselves in the direction of the tensile force in the crack, resulting in the two sides of the fracture surface moving relative to each other (displacement perpendicular to the tensile force).

The experimental results indicate that, contrary to expectations, the matrix strength associated with the initial crack exhibited a clear effect of the test setups, while the effect of the setups on the residual strength and dissipated energy associated with the post-cracking behavior was less pronounced. As previously discussed, Setup I provided the lowest and least reliable matrix tensile strength values, Setup II provided the highest and most reliable values, while the matrix tensile strength values and their standard deviation from Setup III were intermediate high. In terms of residual strength values, the results from Setup I exhibited slightly more favorable outcomes, while those from Setup III exhibited slightly less favorable outcomes. In terms of dissipated energy, the results from Setup II exhibited slightly more favorable outcomes. However, the overall performance was similar for the three test setups in both residual strength and dissipated energy.

In subsequent test series, an alternative approach was explored, whereby specimens were connected to the testing machine via a longer steel reinforcing bar with a smaller diameter at both ends. The relatively flexible reinforcing bars were found to effectively bridge the initial eccentricities and eliminate constraints. Furthermore, the quasi-fixed connections maintain the direction of the tested specimen, thereby limiting the rotation of the test specimen around the fracture surface and the increase of the eccentricities during the testing process. Unfortunately, the results of that study are not directly comparable to those presented here due to the change in materials and the resulting change in recipe. The reason for this change is that the cement and microsilica used, as well as the steel fiber, are no longer manufactured. Consequently, the material and geometrical properties of the successor products are different.

#### 4.2.4. Effect of Notching

**Figure 18:**
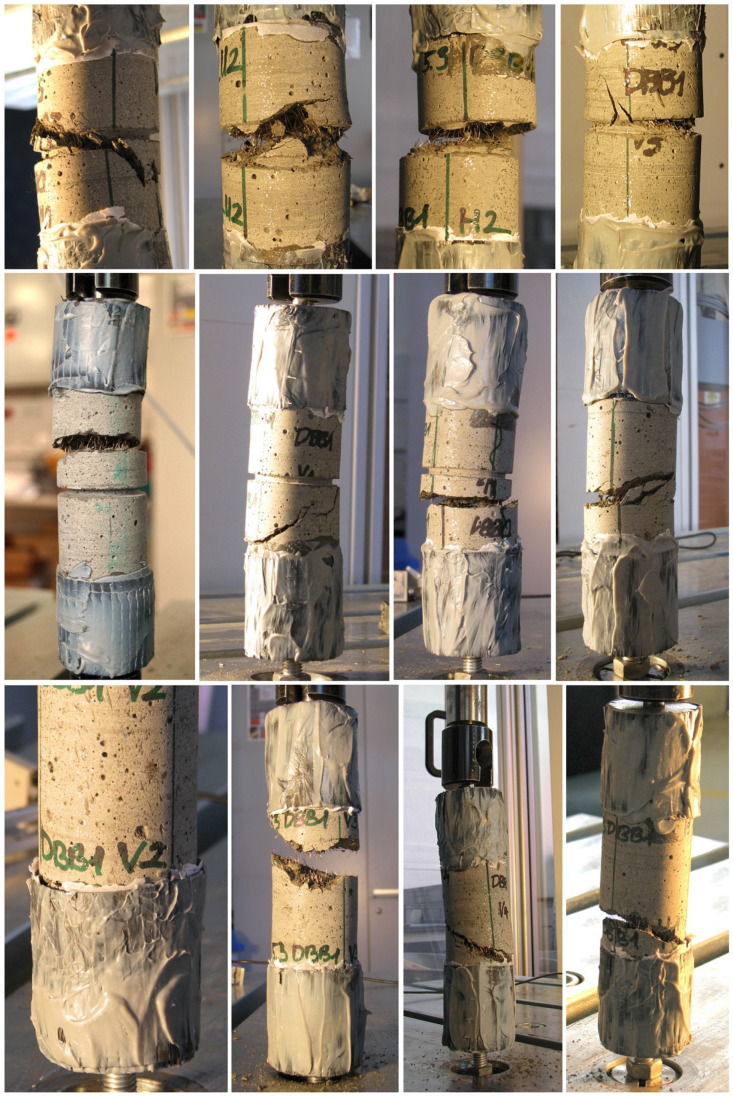
Examples of failure modes and fracture surfaces with and without notch.

One part of Series 1 was performed with unnotched specimens, while the other part was performed with notched specimens. The mean diameter of the unnotched circular specimens was 51 mm, while the mean remaining diameter of the notched specimens was approximately 43 mm. The standard deviation of the diameter of the specimens without a notch did not reach 0.07 mm, while those with a notch were about 0.25 mm. Although the effect of notching is a complex issue due to the resulting stress peaks and the fracture surface may vary with different fiber distributions and orientations (see Figure 18), the effect of notching was considered in this study only with net cross-sections before fracture.

The theoretical advantage of uniaxial tensile testing on unnotched specimens is that the location of crack initiation is not predetermined and the direction of crack propagation is not influenced, so they are truly dependent on the internal distribution of material properties. The advantage of uniaxial tensile testing on notched specimens is that the location of the crack is determined, making it easier to measure and evaluate. In practice, however, unnotched specimens frequently exhibit cracking outside the designated crack measurement area, such as near the specimen’s end or under the bonding area. At higher fiber contents, the fracture surface may exhibit considerable irregularity or diagonal characteristics. Additionally, multiple cracking may occur. In the case of notched test specimens, it is important to note that the predetermined fracture surface may result in failure not occurring at the weakest point of the specimen. Furthermore, the notching does not guarantee that the crack will initiate in the notch or that the fracture surface will propagate through the weakened, well-defined cross-section. These phenomena were all observed in the Series 1 tests. In the second series, the initial two tests were conducted on an unnotched test specimen. In addition to the issues observed in the first series, these specimens exhibited the highest pre-cracking tensile resistance of all the specimens tested in this study, and the sudden release of energy at cracking and the associated dynamic impact were so intense that the sensors attached to the specimen surface slipped, rendering the measurement of crack propagation unusable. The occurrence of the unfavorable phenomena observed in the first series also occurred in the second series serves as evidence that they are not merely a consequence of the test setup utilized in the first series (Setup 1). As a consequence of these negative experiences, further tests of Series 2 and all tests of Series 3 and 4 were performed on notched specimens, and the notch depth was increased. The mean remaining diameter of these specimens was about 38 mm, with a standard deviation of 1 mm. As a result, the cross-sectional area was reduced by approximately half due to the notching, which ensured that the crack usually initiated in the notch and remained within the measured area.

As only the results of the first series are available for evaluation of the effect of notching, it is not possible to draw reliable conclusions. However, the results suggest that unnotched specimens exhibited more favorable matrix tensile strength values prior to cracking (by approximately 25–30% on average), whereas notched specimens exhibited more favorable residual tensile strength and dissipated energy values following cracking (by approximately 20–25% on average).

#### 4.2.5. Effect of the Fiber Content

Series 3 and 4 differed only in the amount of steel fiber used, with 1 percent and 2 percent by volume, respectively. This provided an opportunity to analyze the effect of fiber content on the resulting material properties. The matrix strength values for both series are presented in Figure 14. As previously stated, while the third series provided the highest matrix tensile strength and the fourth series the lowest, the statistical analysis indicates that the two series can be considered practically identical (mean value for both is 7.9 MPa, median value 7.7–7.8 MPa and standard deviation 1.0 MPa). The results support the theory that the fiber content has no significant effect on the matrix tensile strength.

In contrast to the pre-cracking tensile behavior, the expectation for the post-cracking tensile behavior is that the results are more favorable with an increase in fiber content. However, the results of uniaxial tensile tests have not fully confirmed this hypothesis. The highest values of residual tensile strength and dissipated energy were observed in the mixture with 2% fiber, while the lowest value was observed in the mixture with 1% fiber. Furthermore, the mean value of residual tensile strength was slightly higher at the higher fiber content (5.5 MPa vs. 5.2 MPa), and the higher tensile strength was achieved at a smaller crack opening (0.30 mm vs. 0.37 mm). Nevertheless, the dissipated energy values were found to be very similar for both mixtures, with a slight advantage (approximately 5%) for the mixture with lower fiber content. Additionally, the coefficient of variation of the residual tensile strength was less favorable for the mixture with higher fiber content. This indicates that, although the addition of a minimum amount of steel fiber leads to a significant change in the post-cracking tensile behavior, further increases in fiber amount may not always be beneficial. Rather, the distribution and orientation of the added fibers are more important than the amount of fiber itself.

#### 4.2.6. Fiber Orientation and Distribution

Prior to discussing the distribution and orientation of fiber reinforcement, it is essential to clarify that these have not been manipulated in any way in this study. In many cases, the shape or size of the specimen alone has a significant effect on the tensile test results. For instance, in the case of thin plates or small specimens, a substantial proportion of the fibers are oriented parallel to the boundary surfaces during casting, resulting in a planar or even uniaxial arrangement. Moreover, it is a common practice, and several guidelines require that the fresh mix be poured slowly so that the flow in the specimen is longitudinal (e.g., from one end to the other). This also largely determines the orientation of the fibers, which are predominantly in the direction of flow. Furthermore, there are techniques, such as vibration or magnetic effects, for influencing the distribution and orientation of the steel fibers in the still-fresh mixture after casting in a way that is favorable to the expected tensile stresses. The outcomes of tensile tests on specimens produced by these methodologies are considerably more favorable, exhibiting enhanced strength and reduced variability, and the evaluation and comparison of different test series are clearer. Nevertheless, these outcomes can only be utilized in the modeling and design of real structures if the same favorable fiber arrangement and fiber orientation can be guaranteed or if specific experiments are conducted to ascertain the conversion between them. In this study, a different approach has been used, where the aim is to make the production process as similar as possible to the real structural elements so that the results obtained can be representative of the structural elements. In comparison to the dimensions of the prismatic specimen (700 × 150 × 150 mm^3^), the relatively small length of the steel fiber (15 mm) allows for the effect of the side walls to be largely neglected in the majority of the produced specimens. The fresh mixture was poured into the formwork in a vertical direction, simultaneously extending along the majority of the length of the specimen. In contrast to longitudinal pouring, this method results in the most unfavorable fiber arrangement, as will be described later. No subsequent procedures were carried out to influence the fiber orientation or distribution. The cylindrical specimens were then drilled in three directions from the hardened prismatic specimen, as described in Section 2.5.1.

**Figure 19:**
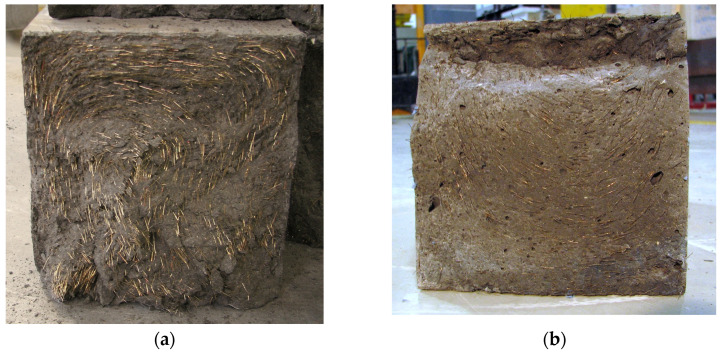
Fiber orientation example shown by the fracture surface of flexural prisms: (**a**) Strong circular fiber orientation with 2 vol% fiber; (**b**) Moderate circular fiber orientation with 1 vol% fiber.

Figure 19a,b depict the fracture surface of prisms resulting from flexural tests, illustrating the fiber orientation as a consequence of this manufacturing process. The circular fiber orientation, often observed in such cases, is clearly visible, resulting from the simultaneous vertical pouring of the mixture, which typically moves in a vertical plane. Such an arrangement of fibers is twofold disadvantageous for the results of tensile tests. Firstly, a smaller proportion of fibers are arranged in the direction of the principal tensile stress trajectories, which is the longitudinal axis of the specimens. Consequently, fewer fibers can bridge the cracks that develop and transfer the tensile forces. Secondly, the fibers arranged perpendicular to the tensile stresses even weaken the given cross-section, providing a natural fracture surface. As a result, the direction of flow of the fresh mixture is often clearly visible from the fracture surface.

**Figure 20:**
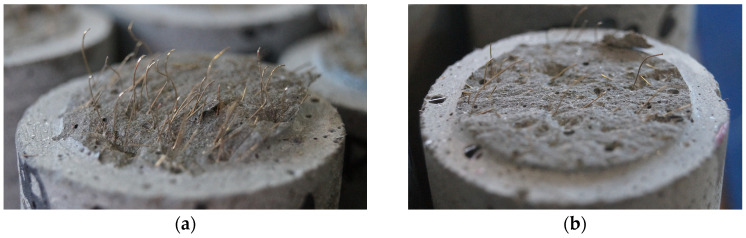
Fracture surfaces with different fiber orientation and distribution: (**a**) Advantageous; (**b**) Disadvantageous.

The differences in fiber orientation and distribution were identified in the uniaxial tensile tests. As illustrated in Figure 20, some specimens exhibited a high density of parallel fibers with a relatively uniform distribution across the fracture surface (Figure 20a), while other specimens exhibited only a few fibers, which were sometimes unevenly distributed across the fracture surface (Figure 20b). This difference became evident in the post-cracking behavior. In the former case, following cracking, the tensile force demonstrated a notable increase, approaching or even exceeding the matrix strength. In contrast, in the latter case, cracking was accompanied by a sudden, pronounced drop in tensile force, followed by either stagnation or a small increase in resistance. In some cases (the three outstanding values of Series 4 discussed earlier), the tensile force and the corresponding tensile stress did not decrease at the time of cracking but instead increased up to a residual tensile stress exceeding 16 MPa. These results demonstrate the potential for optimal or near-optimal fiber orientation, i.e., when the fiber orientation is favorably influenced during casting by the methods described above.

The statistical analysis of the matrix tensile strength values indicates that the direction of measurement has no significant effect. This finding again confirms that the fiber reinforcement has no notable effect on the matrix tensile strength and that such a fine-grained matrix itself can be considered isotropic. When examining each series individually, both the mean and standard deviation of the matrix tensile strength can be considered to be the same in different directions within the confidence interval. From this analysis, the values for Series 1 have been excluded, as it was previously found that the test setup used here does not provide reliable data on matrix tensile strength. However, the remaining 90 values from two different test setups with two different fiber contents from three different test series offer convincing results.

**Figure 21:**
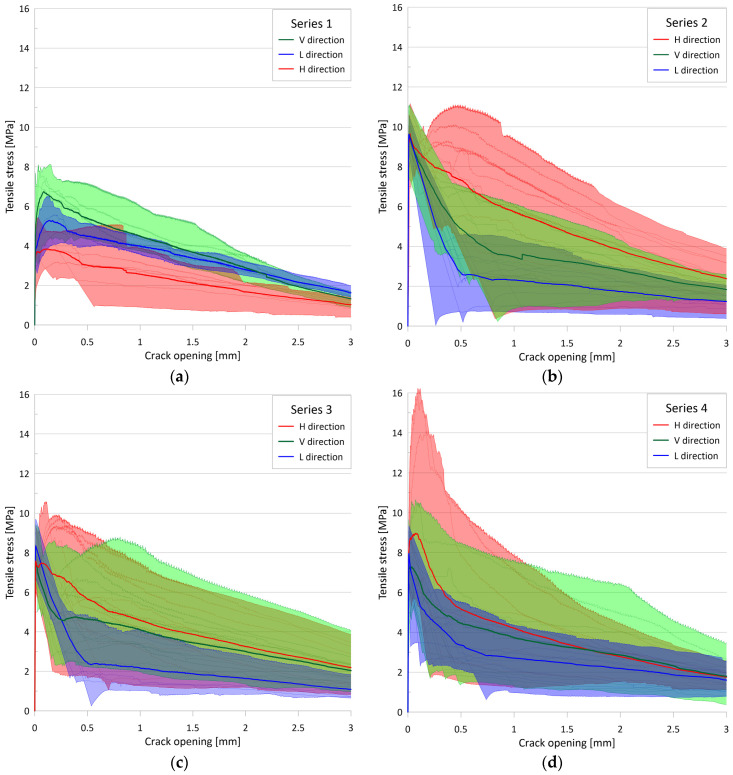
Ranges and mean curves for the tensile stress vs. crack opening: (**a**) Series 1; (**b**) Series 2; (**c**) Series 3; (**d**) Series 4.

In contrast to the pre-cracking behavior, the post-cracking behavior clearly demonstrates the influence of fiber orientation. According to the statistical analysis, the difference between the mean residual tensile strength values (see Table 4) in the unfavorable and favorable directions is two to three times, while for the mean dissipated energy, the values (see Table 5 and Table 6) are one and a half to two times higher in all four test series. Figure 21 visually illustrates the different behavior in the various directions by means of the stress-crack opening curves. All measured curves (in different colors for each direction), their envelope curves for each direction, and the mean curves for each direction are plotted simultaneously. Additionally, the area between the minimum and maximum envelope curves for each direction is filled with the color of that direction.

The plots for Series 2, Series 3, and Series 4 confirm the previously described findings with the measurement data, namely that steel fibers tend to be arranged in the vertical plane for the casting process used, resulting in significantly weaker results for the longitudinal direction (L) than the other two directions in the vertical plane. A comparison of the horizontal (H) and vertical (V) directions in the vertical plane perpendicular to the prism longitudinal axis reveals that the post-cracking curves for the horizontal direction appear to be more favorable, although in many cases, this is reversed for crack openings greater than 0.5 to 1.0 mm. This observation may be explained by the tendency of the vertical steel fibers in a fresh mix with higher consistency to rotate to a stable horizontal position during casting. (This is similar to the phenomenon where steel fibers tend to sink in a mix with a too-fluid consistency, especially in the presence of vibration or other dynamic effects.) The figure for Series 1 gives a somewhat different picture. In this series, the outcomes for the longitudinal direction exhibit more favorable, intermediate results, while the outcomes for the vertical direction are significantly superior to those for the horizontal direction. The more favorable results observed in the longitudinally drilled specimens can be attributed to a slightly different casting method, which differed somewhat from the casting method employed in the subsequent series. In the case of results that are inconsistent with the other series in the vertical and horizontal directions, it cannot be excluded that the prism was rotated by 90 degrees during the drilling process.

In addition to statistical analysis of the numerical values, Figure 21 provides an excellent visual representation of the relationship between the mean curves and between the mean curves and the individual measured curves in each direction as well as the very wide range of tensile stresses resulting from the observed difference between the minimum and maximum values.

Analyzing each curve separately, it can be found that in all four series, there were specimens where the residual tensile strength exceeded the matrix tensile strength, thus their behavior could be considered as strain hardening. The increase in residual strength compared to the matrix strength in these cases was in the range of a few percent to a hundred percent, averaging about 20% for specimens with 1% fiber content and about 40% for specimens with 2% fiber content, resulting in residual tensile strengths of 9.2 MPa and 12.2 MPa, respectively. Given the previous findings on fiber orientation, it is not surprising that none of these curves are from specimens drilled in the longitudinal direction, but 10 are from specimens drilled in the horizontal direction and 8 are from specimens drilled in the vertical direction. (If, however, the horizontal and vertical directions are considered to have been interchanged for the first series, this ratio changes to 12–6.)

For the longitudinal specimens in the first series, and for the longitudinal and horizontal specimens in the third and fourth series, it was indicated which specimens were drilled from the lower and which from the upper half of the prisms. This allows for the evaluation of the fiber distribution for these specimens. Upon evaluation of all relevant specimens collectively, it becomes evident that a degree of steel fiber settling is also observed, as the specimens drilled from the lower part of the prisms provided on average 38% higher residual tensile strength values. When comparing only adjacent specimens, it can be seen that this phenomenon was consistently observed in the longitudinal specimens of the first series and in the horizontal specimens of the third and fourth series. However, the longitudinal specimens of the third and fourth series contradict this. The discrepancy in results can be attributed to the unexpectedly low residual strength exhibited by the bottom-drilled specimens, which averaged only 1.7 MPa for the third series and 2.5 MPa for the fourth series. This contrasts with the expected values of around 4.0 MPa observed in the top-drilled specimens.

#### 4.2.7. Crack Opening at Zero Tensile Resistance

In this study, the tensile stress–crack opening curves obtained from uniaxial tests were used to determine the crack width at which the tensile resistance is eliminated by the complete pullout of steel fibers in the fracture surface. This crack opening value was determined for each specimen at 0 MPa and at the selected stress level of 0.2 MPa. The rationale for the latter is that, from a practical standpoint, it is preferable to set a limit value that is higher than 0 MPa. For specimens of such small dimensions and fracture surfaces, a tensile stress of 0.2 MPa is well within the lowest 1‰ of the measuring range of the loading equipment used. Furthermore, even during the specimen-clamping procedure, for example, a minimum load may be unintentionally introduced, and seemingly insignificant factors such as the self-weight of the specimen and the parts attached to it must be considered. In the uniaxial tensile test, even after complete pull-out, the load cell typically indicated multiples of 10 N with a positive or negative sign. Moreover, the curve is extremely flat in this range. These factors make the determination of the crack opening value at absolute zero load uncertain.

The result of the analysis indicated that the post-peak mean crack opening at 0.2 MPa was 5.8 mm for Series 1, 5.9 mm for Series 2, 6.1 mm for Series 3, and 6.4 mm for Series 4. The standard deviation for these mean values was 0.7–0.8 mm. This compares to a crack opening of 9.3 mm for Series 1, 7.6 mm for Series 2 and Series 3, and 7.9 mm for Series 4 in the case of full pull-out of the steel fibers and loss of tensile resistance. The standard deviation for these values was 2.0 mm, 1.1 mm, 0.6 mm, and 0.9 mm, respectively. When the four series were evaluated collectively, the crack width associated with 0.2 MPa was 6.1 mm, while that associated with the loss of tensile resistance was 7.9 mm, with standard deviations of 0.76 mm and 1.18 mm, respectively.

The outcomes align with those previously reported in the literature. The limit case, where the fibers have no effect on the tensile resistance and can be considered completely pulled out is half of the fiber length (½ × l_f_) [6,9,37,90], which is 7.5 mm for the utilized fiber. It is noteworthy that, in certain instances, crack openings exceeding 9 mm and even 10 mm have been observed in the study with minimal tensile resistance. This is due to the fact that the reported crack openings represent the average of three sensors evenly spaced along the perimeter. If the two parts of the specimen are rotated relative to one another after the fracture surface has formed, the crack may become significantly larger on one side where the pull-out process is complete, while on the other side, there are still a number of steel fibers bridging the crack and capable of exhibiting significant tensile resistance. In such instances, the crack opening value for this late stage of the test averaged over the three measurements is no longer representative of the smaller cross-section remaining and slightly biases the mean value of all measurements upwards. Simplified material models [7,28,80] typically use a quarter of the fiber length (¼ × l_f_) as the crack width where the tensile resistance is reduced to zero. For the fiber used, this is 3.75 mm. This is a low value compared to the measured results, but since these simplified linear models do not take into account the late, flattening part of the curves, the steeply decreasing section associated with the intensive fiber pull-out phase is realistically (though usually conservatively) described. In between these two values is the crack width corresponding to one-third of the fiber length (⅓ × l_f_), which is 5.0 mm for the fiber used, and well represents the crack width where the transition between the steeply declining and flattening final part of the stress–strain curve is usually located.

### 4.3. Discussion on the Flexural Tests

The discussion of the flexural tests in this section focuses mainly on comparing their results with the material properties and constitutive law obtained from the uniaxial tensile tests.

#### 4.3.1. Determination of the Matrix Tensile Strength

In many cases, the elastic limit associated with the matrix tensile strength is determined from the point on the load-deflection diagram where the initial linearity is lost. In many cases, this is easy and quick to determine, while in other cases, it is uncertain to define. The determination of the elastic limit based on crack measurement is more reliable and accurate. Compared to conventional crack measurements using strain transducers or displacement transducers, it is even more advantageous to monitor the surface of the test specimen using a refined optical method such as digital image correlation (DIC). In this case, the crack cannot develop outside the sensor’s measurement range. Unlike surface-mounted sensors, it is not sensitive to impact effects that may occur during loading, elastic elongations and deformations associated with cracks can be separated, and it is even possible to measure each crack separately instead of measuring the average value on the base length of the sensor [73,76].

In the flexural tests of the prismatic specimens, the average flexural tensile strength determined for the matrix was found to be 9.5 MPa, while in the flexural tests of thin plates, the average flexural tensile strength determined for the matrix was 12.8 MPa. For the conversion between the value of the mean flexural tensile strength (f_ctm,fl_) determined for the outer fiber of the bent specimens and the mean value of the tensile strength (f_ctm_) determined by the uniaxial tensile test, several guidelines require the application of a scale factor (α_fl_):

f_ctm_ = α_fl_ × f_ctm,fl_, (3)

where the alfa can be calculated with the following equation:

α_fl_ = k × h^0.7^/(1 + k × h^0.7^). (4)

The coefficient k was given by CEB-FIP ModelCode 1990 [91] with a value of 0.06 to take into account the scale effect for normal concrete. *fib* ModelCode 2010 [23] recommends using the same expression with a note that, for high-strength concrete, it should be replaced by a value of k lower than 0.06 due to the increased brittleness. The AFGC/SETRA [28] and AFGC [7] recommend the formula with the use of the k coefficient of 0.08 for UHPFRC. The French standard for UHPFRC [8] also employs the value k = 0.08, with the note that, for strain-hardening UHPFRC, the value may be recalibrated, resulting in a coefficient k that is generally greater than 0.08. Applying the relationship from Equation (4) and the coefficient k = 0.08 according to French specifications [7,8,28], the scale factor for the bent prisms used is 0.727, resulting in a calculated uniaxial tensile strength of 6.9 MPa. The same relationship for the bent thin plates gives a scale factor of 0.464, which would give a uniaxial tensile strength of 5.9 MPa. If the relationship derived from fracture mechanics considerations is calibrated to the experiments performed, a value of k = 0.15 for both bent prisms and bent plates would give a uniaxial tensile strength of 7.92 MPa with scale factors of 0.83 and 0.62, respectively. Nevertheless, the values presented here are intended to provide information on the experiments conducted, as the scope of this study is not adequate for a reliable determination of the coefficient k, which requires a larger number of experiments and further investigations. Furthermore, the French UHPFRC specifications require the use of the scale factor only for flexural tests on prisms. For flexural tests on thin plates, the value of the (uniaxial) tensile strength is equated to the value of the flexural tensile strength determined from the point on the moment curve corresponding to the elastic limit (or loss of linearity). The results of the tests demonstrate that the proposed approach yields a tensile strength of 12.8 MPa, whereas the application of the scale factor (and k = 0.08) results in a tensile strength of 5.9 MPa. In comparison to the outcomes of the other tests, the former deems to be too high, while the latter appears to be insufficiently low. In contrast, the value of 7.9 MPa resulting from the uniaxial stress–strain relationship obtained by inverse analysis is a more realistic representation of the material’s matrix tensile strength.

In the absence of experimental data on the matrix tensile strength, it can be estimated from the compressive strength for preliminary design by using one of the following simple expressions:

f_ctm_ ≈ 0.3 × (f_ck_)^2/3^, (5)

f_ctm_ ≈ 0.68 × (f_cm_)^1/2^, (6)

where f_ck_ is the characteristic, while f_cm_ is the mean compressive strength measured on standard cylinders at 28 days. Equation (5) follows the European conventions and is offered by the Eurocode 2 [92] for normal strength concrete (NSC), by RILEM [93] for fiber-reinforced concrete (FRC), and by the Austrian Directive for UHPFRC [10], while Equation (6) is calibrated to align with the terminology that is customary in the United States [42]. By applying the relationship

f_cm_ ≈ f_ck_ + 8 MPa, (7)

which can also be utilized for preliminary design under the UHPFRC specifications [6,10], the two terms can be compared. The two expressions yield the same tensile strength value of 8.74 MPa at a mean compressive strength of f_cm_ = 165.4 MPa. For lower compressive strengths, Equation (6) gives slightly higher tensile strength values, whereas for higher compressive strengths, Equation (5) gives slightly higher tensile strength values. For the compressive strength determined for the mixture without steel fibers used in the study, Equation (5) gives 7.94 MPa, and Equation (6) gives 8.17 MPa. The tensile strengths obtained from the first (Equation (5)) and second (Equation (6)) relationships for the mixture containing 1% fiber are 8.60 MPa and 8.64 MPa, respectively, and for the mixture containing 2% fiber are 8.77 MPa and 8.76 MPa, respectively. The values determined from the two relationships are reasonably close for estimation and are in agreement with the measured results for the tensile strength of the matrix.

#### 4.3.2. Comparison with the Uniaxial Tests

**Figure 22:**
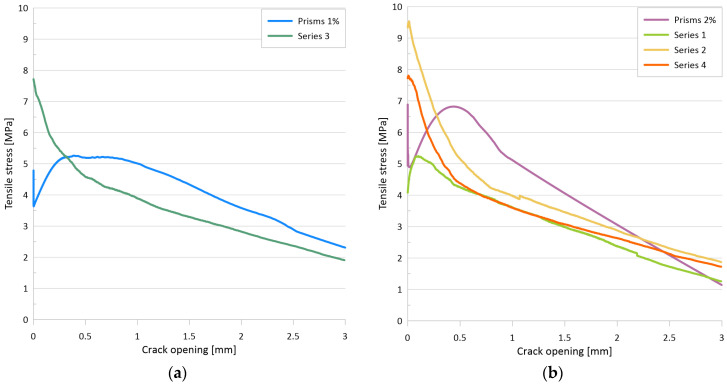
Comparison of results of the flexural and uniaxial tests: (**a**) 1% mixture; (**b**) 2% mixture.

Figure 22 presents a comparison of the uniaxial tensile stress–crack opening curves derived from the flexural tests by inverse analysis and those directly obtained from the uniaxial tensile tests, separately for the UHPFRC mixture with 1% steel fiber content (Figure 22a) and the UHPFRC mixture with 2% steel fiber content (Figure 22b). The figures show the average curve for each series of tests. The difference in matrix strength at the beginning of the curves is striking. The reasons for this have been discussed previously. However, the most significant difference is observed in the character of the curves. In the case of the curves derived from the flexural tests, the cracking of the specimens always led to a significant and immediate drop in tensile stress (with only a slight increase in crack opening), followed by a gradual increase in tensile stress in the investigated mixtures, parallel to the activation of the steel fibers, with a local peak at approximately 0.5 mm, after which the stresses slowly decreased. In contrast, the average curves obtained from the uniaxial tensile tests exhibit a nearly monotonically non-increasing trend, with the exception of the average curve from Series 1, which is discussed in more detail in the preceding Section 4.2.3. The background is that some of the curves obtained from the uniaxial tensile tests exhibited a sudden, significant cracking accompanied by a steep drop in tensile resistance when the tensile strength of the matrix was reached, and only a moderate rebuilding afterward, while others showed hardening behavior after cracking and then a gradual decrease after reaching a local maximum. The average of these curves with opposite trends resulted in the characteristic of the average curve presented in the figure, which shows a gradual decrease with increasing crack width. Overall, it can be observed that the tensile stress values derived from the uniaxial tensile tests exhibit a higher level of stress in the range below approximately 0.3 mm, while the tensile stress values derived from the flexural tests exhibit a higher level of stress in the range above approximately 0.3 mm. Moreover, they indicate roughly the same values again in the range of the crack widths of 2.5–3.5 mm. Nevertheless, these differences between the curves are of minimal consequence in practice, as they are within the confidence range of the results obtained from these tests, the values for both the serviceability limit states (SLS) and the ultimate limit states (ULS) are close, and the area under the curves yields similar outcomes.

#### 4.3.3. Determination of the Tensile Strain

**Figure 23:**
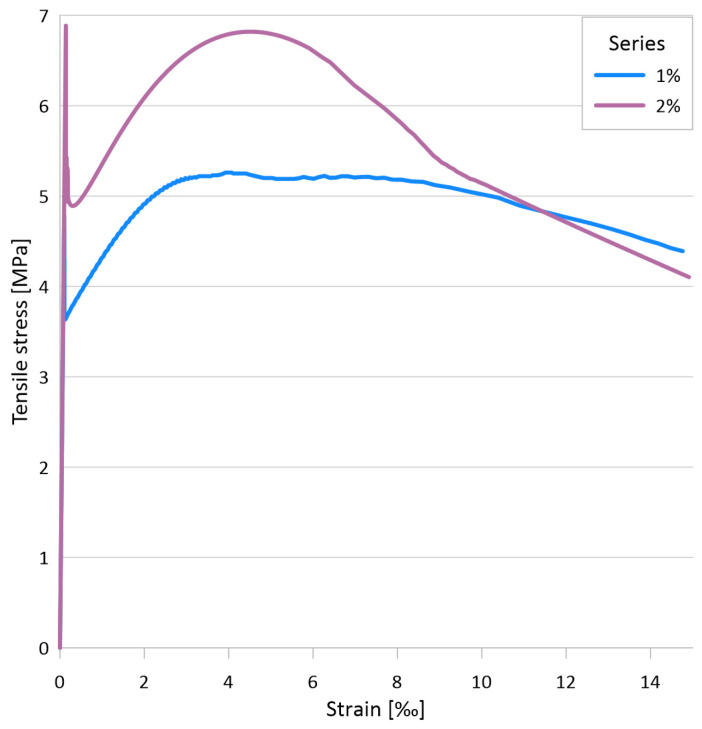
Tensile stress–strain relations from the flexural tests on prismatic specimens.

For strain-softening or low strain-hardening UHPFRC, flexural tests on prismatic specimens are based on CMOD measurements, and the uniaxial tensile stress versus crack opening relation is determined by means of inverse analysis. However, to determine the constitutive law, the tensile stress–strain relationship is required. For this purpose, UHPFRC guidelines [7,80] recommend the use of the following relationship:

ε = f_ct_/E_cm_ + w/l_c_, (8)

where ε is the tensile strain, E_cm_ is the secant modulus of elasticity, and l_c_ is the so-called characteristic length. Based on fracture mechanics, the French specifications [7,80] suggest the following simple relationship for rectangular cross-sections:

l_c_ = 2/3 × h, (9)

where *h* is the height of the cross-section. Using this procedure and the tensile stress–crack opening curves shown in Figure 8b, the tensile stress–strain curves shown in Figure 23 could be determined.

For flexural tests on pronounced strain-hardening UHPFRC prisms and for flexural tests on thin plates, this procedure is not necessary because the very fine crack pattern allows direct determination of the stress–strain curves (see Figure 10b).

#### 4.3.4. Comparison with Simplified Models

**Figure 24:**
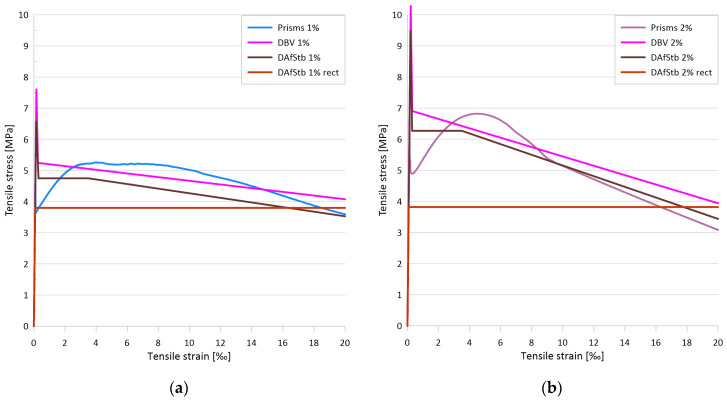
Derived and simplified tensile constitutive laws: (**a**) 1% mixture; (**b**) 2% mixture.

The stress–strain relationships derived from the flexural tests using inverse analysis can be compared with the simplified stress–strain relationships defined by different guidelines. In this study, the simplified procedures proposed by the German Concrete and Construction Technology Association (DBV) and the German Committee for Structural Concrete (DAfStb) were examined, as the test specimens used in the study and their test procedure were in accordance with the requirements of these guidelines. Figure 24 presents a comparison between the average tensile stress–strain curve derived by inverse analysis, the simplified tensile constitutive law defined by the DBV’s FRC guideline [34], and the simplified tensile constitutive laws defined by the DAfStb’s FRC guideline [29], separately for the UHPFRC mix with 1% steel fiber (Figure 24a) and 2% steel fiber (Figure 24b). The DAfStb’s guideline presents two different simplified models: one consisting of four linear sections and one describing perfectly elastic-ductile behavior (‘rectangular’). To evaluate the comparison, it is useful to note that the DBV’s FRC guideline was issued earlier, in 2001, while the DAfStb’s FRC guideline was first issued in 2010 and can be considered an evolution of the DBV’s guideline. Moreover, it is also important to note that neither of these guidelines applies to high-strength concrete (HPC), ultra-high-performance concrete (UHPC), and self-consolidating concrete (SCC), therefore, their application to UHPFRC is questionable. However, the results suggest that they may be suitable. Each of the curves begins with a linear elastic section (barely visible in the figure), which continues up to the specified matrix tensile strength. The exception to this is the perfectly elastic-plastic model, which continues in a horizontal section after reaching the tensile strength value considered valid for the entire cracked range up to 25‰. For the remaining models, the cracking is followed by a significant drop in resistance, which is then followed by a single, linearly decreasing section for the DBV model and a horizontal and a linearly decreasing section for the DAfStb model. The points of the models presented in the figure are determined from the recorded applied load-deflection curves for each model, based on the areas under the curves (absorbed energy) for the DBV model and on the applied load values associated with specified deflection values for the DAfStb model. For the investigated mixtures, the DAfStb model yielded more conservative results than the DBV model for the entire valid range. A comparison of the two DAfStb models revealed that the perfectly plastic model provided lower stress values than the four-line model for a large part of the range (for elongations less than 16–18‰). When the simplified models are compared with the curves derived by inverse analysis, the largest and most important difference is observed after the cracking. At this stage, the drop of the post-cracking tensile stress is significantly higher than that described by the models. Furthermore, the subsequent activation of the steel fibers results in a larger stress increase than that predicted by the models. Consequently, the average of the values and the areas under the curves for the entire range yield comparable results. The models exhibit greater similarity in their characteristics to the average curves derived from uniaxial tensile tests, prompting the question of whether the stress drop following cracking in the flexural tests is a real physical phenomenon or a consequence of the testing method and its evaluation. In addition, it can be observed that the perfectly ductile model calculated for the mixtures with 1% and 2% steel fiber content provided almost the same values (constant stress level of 3.80 MPa and 3.82 MPa), confirming the observation made in the uniaxial tensile tests (see in Section 4.2.5) that the mixtures tested can be considered practically identical based on the statistical evaluation when calculated over the entire range. Overall, the simplified tensile constitutive laws model of the UHPFRC mixtures investigated surprisingly well, with the multi-linear model of DAfStb appearing to be particularly suitable for describing them.

#### 4.3.5. Crack Opening–Deflection Relationship

The study further investigated the relationship between the deflection and the crack opening in the flexural experiments. Considering that multiple cracking can always be expected in the case of unnotched steel-fiber-reinforced UHPC specimens subjected to a four-point bending test (and many times also in the case of notched specimens subjected to a three-point bending test), the applied crack opening is the sum of the CMOD values (and elastic elongation) measured along the middle third of the prism (between the two load application points). Linear regression analysis was performed using the ordinary least squares method and resulted in the following linear relationship:

(10) $$\begin{array}{ll} w(\delta )\;=\;0 & \mathrm{if}\;\delta \;\leq \;0.15\;\mathrm{mm},\\ w(\delta )\;=\;1.18\;\times \;\delta \;-\;0.18 & \mathrm{if}\;\delta \;>\;0.15\;\mathrm{mm}. \end{array}$$

The presented linear relationship exhibits a high correlation with the test results (coefficient of determination r^2^ = 0.9993). However, to achieve an even more accurate relationship, multiple linear regression analysis was also performed, and the following cubic polynomial expression showed optimal correlation (coefficient of determination R^2^ = 0.9999), considering also its applicability in practice:

(11) $$\begin{array}{ll} w(\delta )\;=\;0 & \mathrm{if}\;\delta \;<\;0.13\;\mathrm{mm},\\ w(\delta )\;=\;-0.009\;\times \;\delta^{3}\;+\;0.08\;\times \;\delta^{2}\;+\;\delta \;-\;0.13 & \mathrm{if}\;\delta \;\geq \;0.13\;\mathrm{mm}. \end{array}$$

**Figure 25:**
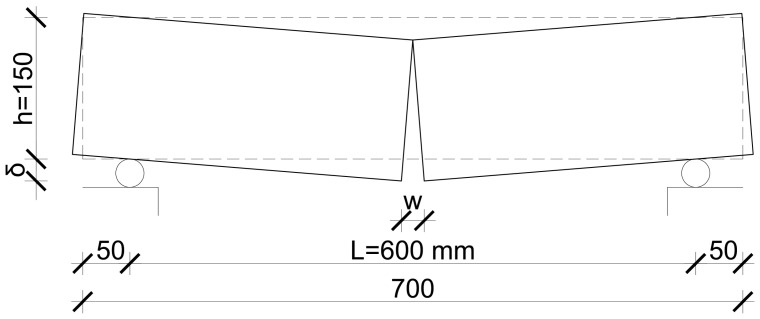
Simplified representation of the deformed specimen geometry.

The polynomial relationship provides a more accurate result, but its range of applicability is limited to approximately 6.2 mm deflection (equivalent to a 7.0 mm crack width). In contrast, the linear relationship demonstrates good agreement over the full measured range. In these relationships, the measured mid-span deflection in millimeters, designated by the symbol *δ*, is expressed in conjunction with the crack opening in millimeters, designated by the symbol *w* (see Figure 25). As indicated by the expressions, the crack opening is zero up to a deflection of 0.15 mm and 0.13 mm, respectively. These deflections (where the test specimen cracks) were typically reached by the specimens tested at applied loads between 55 kN and 70 kN, which corresponds to a flexural tensile strength of 9.8–12.4 MPa. In consideration of the previously discussed scale factor of 0.727, the uniaxial tensile strength of the matrix is calculated to be between 7.1 MPa and 9.0 MPa, which is consistent with the previously discussed values.

**Figure 26:**
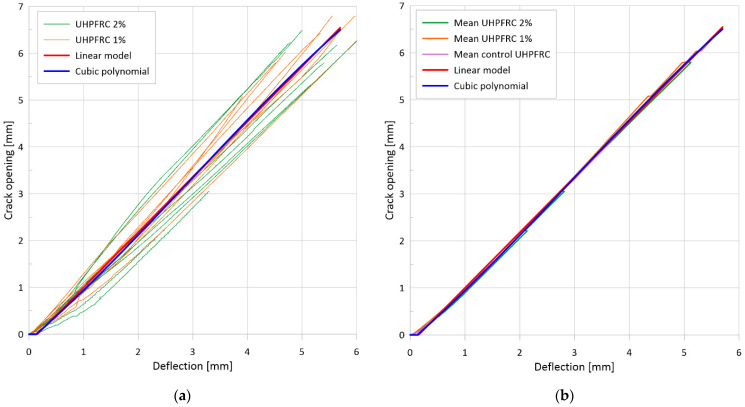
Results of the regression analysis for the prisms (crack opening–deflection relationship: (**a**) with single curves; (**b**) with mean curves and with an additional control series).

The regression analysis was based on data from eight experiments on specimens with a 2% steel fiber content and nine experiments on specimens with a 1% steel fiber content. Figure 26a depicts the empirical data and the corresponding regression curves. Although the individual measured curves exhibit a notable divergence from the regression curves, the mean curves (Figure 26b) demonstrate a satisfactory visual alignment. The data presented in the figure indicates that the average curves are not influenced by the steel fiber content utilized. To verify the validity of the derived equations, it was also compared with the results of another series of tests, where not only a different type of steel fiber was utilized, but also the matrix formulation, components, and production technology were significantly different. This series is designated as “Mean control UHPFRC” in the figure and is located virtually entirely behind the regression curve. The study indicates that the derived relationship is applicable to typical UHPFRC mixtures (in this study, there was a mean cylindrical compressive strength between 160 and 170 MPa, a matrix strength around 8–10 MPa, and a modulus of elasticity between 47 and 51 GPa), using the same specimen geometry and experimental setup.

Similar relationships have been presented previously for flexural testing of fiber-reinforced prisms. The equations proposed by EN 14651 [18] and RILEM TC 162-TDF [17] provide a relationship for notched normal-strength concrete specimens of similar dimensions in a three-point bending setup. The relationship proposed by EN 14651 is as follows:

δ(w) = 0.85 × w + 0.04.(12)

By rearranging the equation for the crack opening, the following relationship is obtained:

w(δ) = 1.1765 × δ − 0.047.(13)

The relationship proposed by the RILEM TC 162-TDF is as follows:

w(δ) = 1.18 × δ − 0.0416.(14)

For the flexural testing of UHPFRC, the NF P 18-470 [8] provides a similar relationship for smaller, notched specimens in a three-point bending setup, described in Equation (15):

w(δ) = 4/3 × 0.9 × (δ − δ_0_). (15)

In this equation, $\delta_{0}$ is the deflection value corresponding to the first crack (end of the elastic region). As previously explained, this value was approximately 0.15 mm in the study. By applying this value and rearranging the equation to a similar form as before, the relationship Equation (16) is obtained:

w(δ) = 1.2 × δ − 0.18.(16)

The close similarity of the above relationships (Equations (13), (14), and (16)) with the one derived in the present study (Equation (10)) lends further support to the reliability of the relationships derived from the study. Moreover, it suggests that the relationships are applicable not only to the testing of unnotched specimens in a four-point bending setup but also to the testing of notched specimens in a three-point bending setup. Furthermore, the relationships do not appear to be particularly sensitive to the specimen dimensions. However, it is essential to highlight that the L/h ratio of the span length L to the specimen height h was consistently between 3.3 and 4.3 across all tested cases, with a typical value of 4. The value of the coefficient corresponding to the deflection is also approximately 1.18 for specimens of similar dimensions, while the constant part of the relationship is related to the appearance of the first crack, thereby implying that a value of approximately −0.18 mm is also expected for materials with similar tensile strengths.

A similar study was conducted for the flexural tests of the plate specimens. However, in the case of the bending of UHPCFR plates, a dense network of numerous microcracks is formed, and thus the average value of the surface elongation resulting from these cracks (and from the elastic elongation) is used instead of the crack width. Consequently, in this case, the deflection–strain relationship was investigated, and the simple linear regression analysis resulted in the following simple equation:

ε(δ) = 0.715 × δ.(17)

The linear relationship demonstrated a satisfactory correlation with the measured results. However, to achieve an even more precise estimation, a multiple linear regression analysis was also conducted. Based on the analysis, the following second- and third-order terms exhibited the optimal results, while maintaining the simplicity required by the practice:

ε(δ) = 0.003 × δ^2^ + 0.67 × δ, (18)

ε(δ) = −0.00022 × δ^3^ + 0.0092 × δ^2^ + 0.63 × δ. (19)

**Figure 27:**
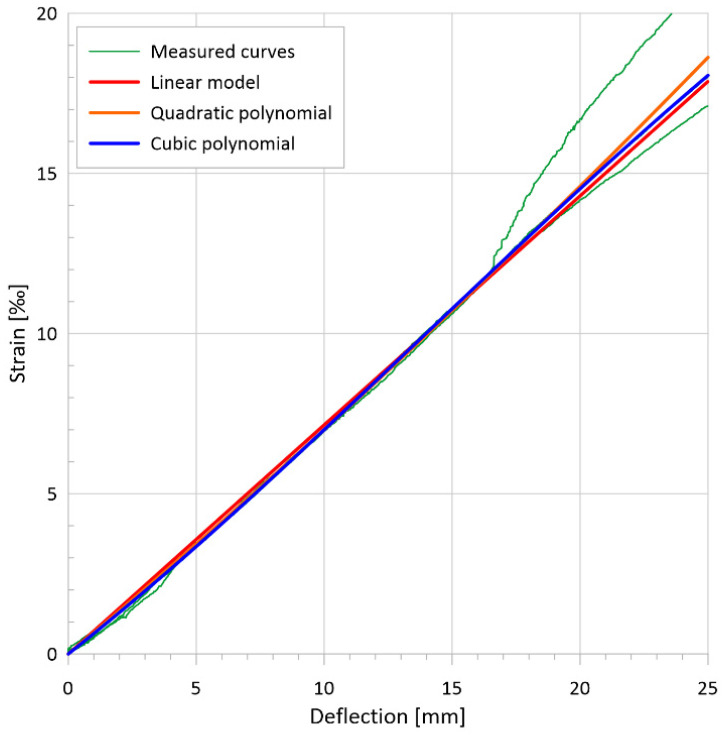
Results of the regression analysis for the plates (crack opening-deflection relationship).

In these relationships, the mid-span deflection in millimeters, designated by the symbol *δ*, is expressed in conjunction with the average strain of the bottom surface between the load introduction points in promil, designated by the symbol *ε*. The range of applicability is limited to approximately 26–27 mm deflection (equivalent to 19–20‰ strain value). Figure 27 depicts the measured curves and the calculated regression curves. (The measured curves are mainly behind the regression curves due to the high correlation). The measured strain–deflection curves exhibit a narrow range of variation relative to each other for the majority of the measured range. However, at approximately 17 mm of deflection (at a strain of about 12‰), the individual measured values exhibit a notable increase in dispersion. This is the point at which the final, decisive crack is formed (crack localization). It is also at the post-peak, descending section of both the applied load–deflection (or equivalent flexural stress–deflection) and uniaxial stress–strain curves (see Figure 10a,b). Thus far, the coefficient of determination R^2^ has been found to be 0.9991 for the first-degree (linear) regression curve, 0.9998 for the second-degree (quadratic) regression curve, and 0.9999 for the third-degree (cubic) regression curve. Further investigation is required to determine which of the three estimations offers the most reliable correlation for the final part of the relationship. However, the relatively small effect of their difference means that they are already applicable to practice.

### 4.4. Discussion on the Compression Tests

**Figure 28:**
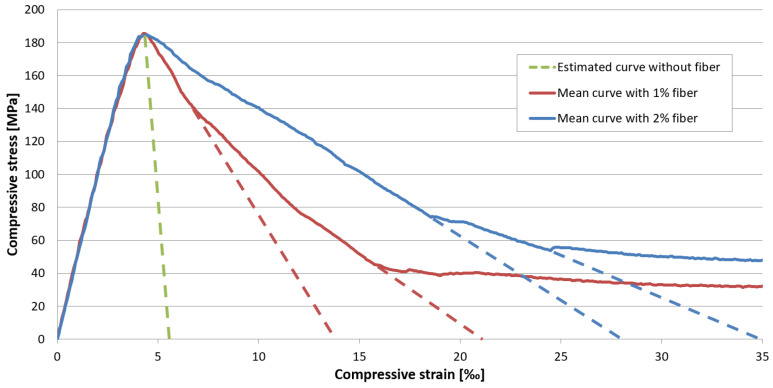
Remarks on the compression curves.

The section discusses the compressive stress–strain curves derived from compression tests on the cubes. In Figure 28, the green dashed line indicates the typical post-peak phase of a mixture without steel fiber (in cases where complete rupture of the specimen does not occur when the peak value is reached, it can be measured). The red and blue dashed lines are employed to characterize the post-peak behavior of mixtures reinforced with 1% and 2% by volume steel fiber, respectively. Two distinct approaches were employed to represent the declining phases of the curves for both mixtures: a linear and a bilinear. The significance of these simplified curves lies in the manner of compressive failure of the fiber-reinforced UHPC, which is unusual for materials with brittle behavior such as concrete. In contrast to the unreinforced UHPC, the steel-fiber-reinforced UHPC does not exhibit fracture behavior after reaching the peak value but exhibits a high degree of plastic deformation by microcracking. Consequently, during compression testing, the shape of the specimen after reaching the peak value becomes less and less like a regular cube. Initially, it becomes rectangular with transverse deformations on the sides and then more and more like a plate. Concurrently, the slope of the stress–strain decreases, then becomes approximately horizontal, and finally, the stress values begin to increase again with higher deformations (this part is not shown in Figure 28). Therefore, for the determination of the real material characteristics, the curve shall be considered only until its shape remains approximately cubic and it reaches the roughly horizontal phase at the most, and the simplified curves marked with dashed lines shall be used instead of the measured curves. The areas between the green dashed curve (non-reinforced UHPC) and the curves for UHPC mixtures reinforced with 1% and 2% steel fiber represent the values of the energy absorbed and thus the efficiency of the fiber reinforcement on the compression side.

### 4.5. The Constitutive Law Derived

**Figure 29:**
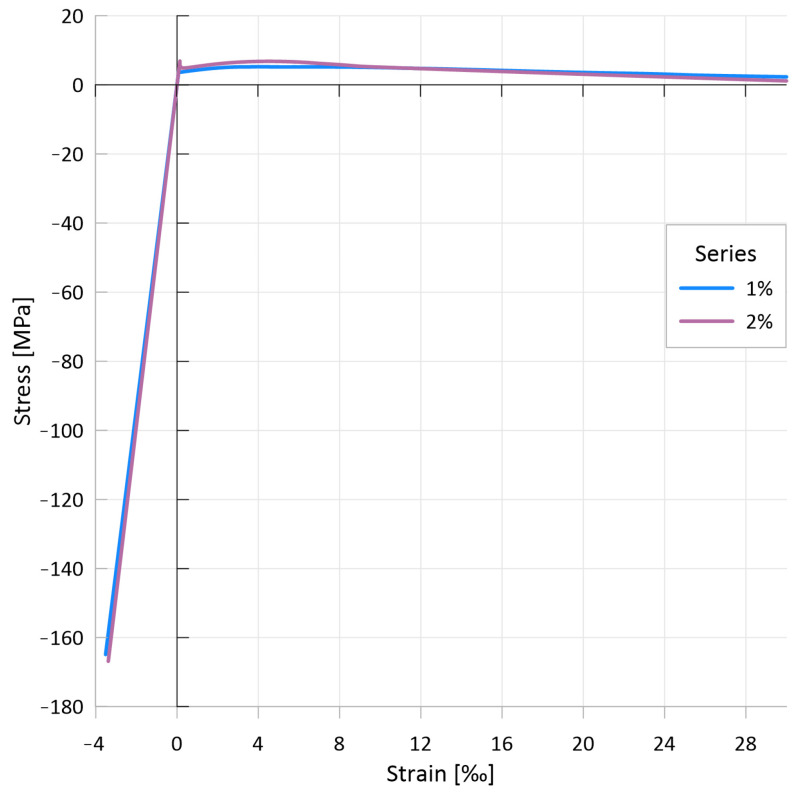
Constitutive law for both mixtures.

The previously derived tensile stress–strain relationship is combined with the compressive stress–strain relationship to derive the overall constitutive law for the mixtures. Figure 29 illustrates the constitutive law for the two UHPFRC mixtures (with 1% and 2% steel fiber) investigated in this study. For the purposes of this illustration, the tensile side is considered to be positive, while the compressive side is considered to be negative. For the tensile side, the stress–strain relationships derived by inverse analysis from flexural tests are presented. For the compressive side, the relationship is presented as a linear elastic relationship up to the measured compressive strength with no plastic or softening part. The figure illustrates the different nature of the tensile and compressive sides of the UHPFRC constitutive law. On the one hand, there is a huge difference of approximately twenty times the magnitude between the stress values obtained on the compressive and tensile sides. On the other hand, while the compressive side allows for only moderate (3.5‰ to 4.5‰) strains without any plastic behavior to be considered, the tensile side is capable of very large (limited by various design guidelines to 25‰ or 50‰) plastic deformations and ductile behavior by means of cracks bridged by steel fibers. The presented constitutive law can be employed directly or in a simplified manner for structural design purposes and can be used to calculate the behavior of UHPFRC in tension.

## 5. Conclusions

In this study, the constitutive laws of a self-developed ultra-high-performance fiber-reinforced cementitious composite were derived through the application of a variety of laboratory tests, with a particular focus on the material’s behavior in tension. Two different mixtures of 1% and 2% by volume steel fiber content were investigated through a splitting tensile test, a uniaxial tensile test using three different test setups, and a flexural test on prismatic specimens and on thin plates. Additionally, the compressive properties and modulus of elasticity of the mixtures were also determined.

- The study demonstrated that the splitting tensile test can provide a reliable estimation of the matrix tensile strength, both for UHPC without fibers and for UHPFRC with low fiber content. Furthermore, the conversion factor between the uniaxial and splitting tensile strengths should be taken as at least 1.0.
- An investigation of the various test setups revealed that Setup I yielded unreliable results for the matrix tensile strength. In contrast, Setup II yielded matrix strength values around 10 MPa. Other experiments, such as the uniaxial tensile test using Setup III and flexural tests and splitting tensile tests, yielded strength values around 8 MPa.
- Upon examination of the post-cracking behavior of the uniaxial tensile tests, it becomes evident that there is a considerable disparity between the experimental results. The difference between the minimum and maximum strength values reached a factor of 10, while for the energy absorption capacity, it was approximately 5. The results of the flexural tests also showed a significant variation, but much smaller: the extreme results were generally within a range of 20–30%, although occasionally the difference was close to 40%. The results also indicated that for excellent post-cracking behavior, it is not enough to have an excellent strength value, but it is also necessary to maintain this high value over a wider cracking range.
- The findings indicated that the significance of the test setup in the post-cracking phase of the uniaxial tensile tests was comparatively less pronounced. However, the influence of fiber orientation and fiber distribution emerged as a dominant factor, and the precise way in which the fresh mix is poured is of critical importance.
- The uniaxial tensile tests have demonstrated the practical limitations associated with specimens without notches. Moreover, the approximately 30% reduction in the cross-sectional area achieved with a 4 mm notch has been found to be insufficient to reliably influence crack location. Consequently, a cross-sectional reduction of approximately 50% achieved with a 6.5 mm notch was identified as a more suitable solution.
- The flexural experiments suggest that the scale factor for the conversion between the matrix flexural tensile strength and uniaxial tensile strength might be increased, at least for the experiments investigated. Furthermore, for flexural tests on thin plates, the proposed value of 1.0 for the scale factor overestimates the matrix tensile strength, but the value determined by inverse analysis is a satisfactory approximation.
- A comparison of the direct and indirect tensile experiments investigated revealed that in the range of small crack widths, the derived tensile stress–crack opening curves have different characteristics, yet yielded comparable outcomes from a practical standpoint. the investigated simplified tensile laws developed for fiber-reinforced NSC, especially the DAfStb’s multi-linear approach, appeared to be applicable to UHPFRC as well.
- The study investigated the relationship between the deflection and crack opening, as well as the deflection and strain values in flexural experiments. The relationships obtained for typical UHPFRC mixtures and the given test setup and test specimen geometry were found to be invariant with respect to the material composition utilized or the type and quantity of steel fibers employed. The presented expressions are straightforward to employ in practice on mean values of at least six experiments and permit the derivation of crack openings and elongations on the tensile side from measurements of mid-field deflections alone, rather than complicated and unclear elongation (crack or strain) measurements. The crack opening and strain values thus obtained from the deflection can be utilized either directly or as input to inverse analysis to derive the constitutive law (uniaxial stress–strain relationship).

The results presented in this study were based on bending tests on molded prisms and plates and on uniaxial tensile tests on drilled specimens. However, the study did not address the determination of the relationship between the results obtained from uniaxial tensile tests on cast specimens (e.g., according to [9,27]) and those obtained from uniaxial tensile tests on drilled or cut specimens with different dimensions.

## Figures and Tables

**Table 1:** Testing program of the uniaxial tests.

Test Series	Direction	Test Setup	Fiber Content	Number of Specimens
Series 1	H	Setup I	2 vol%	6
	V			6
	L			3
Series 2	H	Setup II		12
	V			11
	L			8
Series 3	H	Setup III	1 vol%	12
	V			12
	L			7
Series 4	H		2 vol%	11
	V			11
	L			8

**Table 2:** Results of the splitting tensile tests.

Fiber Content [vol%]	Splitting Tensile Strength [MPa]	Mean [MPa]	Standard Deviation [MPa]	Coefficient of Variation [%]
0	9.56	7.87	0.96	12.2
	6.74			
	7.96			
	6.99			
	7.43			
	8.53			
1 ^1^	8.34	8.07	0.79	9.8
	8.88			
	7.00			
1 ^2^	13.21	14.08	1.02	7.3
	13.51			
	15.51			
2	16.23	16.15	1.20	7.4
	14.65			
	17.57			

^1^ Measurement was taken at the first significant drop in load (local maximum), ^2^ Measurement was taken at the maximum point of the loading curve.

**Table 3:** Matrix tensile strength (f_ct_) from uniaxial tensile tests (in MPa).

Test Series	Direction	Mean of f_ct_	Median of f_ct_	St. Deviation of f_ct_	Min. of f_ct_	Max. of f_ct_
Series 1	H	3.87	3.79	1.16	2.05	5.43
	V	6.54	7.31	1.75	3.28	8.10
	L	4.20	4.28	0.95	3.00	5.33
Series 2	H	9.99	10.03	0.58	8.92	11.17
	V	9.71	9.67	1.00	7.54	11.11
	L	9.63	9.45	0.57	8.77	10.57
Series 3	H	8.00	7.86	1.15	6.12	10.59
	V	7.56	7.44	0.74	6.60	9.43
	L	8.37	8.43	1.08	6.72	9.69
Series 4	H	7.92	7.69	0.96	6.59	9.31
	V	7.68	7.71	1.07	6.11	9.65
	L	8.00	8.11	0.99	6.52	9.36

**Table 4:** Post-cracking residual tensile strength (f_cf_) from uniaxial tensile tests (in MPa).

Test Series	Direction	Mean of f_cf_	Median of f_cf_	St. Deviation of f_cf_	Min. of f_cf_	Max. of f_cf_
Series 1	H	3.38	3.50	1.40	1.00	5.11
	V	6.53	6.55	1.16	4.45	8.12
	L	5.87	5.58	1.52	4.18	7.86
Series 2	H	7.44	8.59	3.05	0.90	11.11
	V	4.23	3.68	2.43	1.11	8.47
	L	2.51	2.58	1.25	0.76	4.64
Series 3	H	6.34	5.80	2.75	1.80	9.94
	V	5.35	5.16	2.36	2.51	8.81
	L	2.62	1.96	1.46	1.07	5.06
Series 4	H	7.44	5.46	5.47	1.88	16.23
	V	5.28	5.05	3.17	1.52	10.65
	L	3.33	3.02	1.44	1.10	6.18

**Table 5:** Dissipated energy up to w = 2 mm (W_f,2_) from the uniaxial tensile test (in kJ/m^2^).

Test Series	Direction	Mean of W_f,2_	Median of W_f,2_	St. Deviation of W_f,2_	Min. of W_f,2_	Max. of W_f,2_
Series 1	H	5.31	5.54	1.51	2.98	7.71
	V	9.14	8.54	1.39	7.94	11.85
	L	7.91	7.58	0.61	7.39	8.76
Series 2	H	11.98	12.38	3.83	5.59	17.82
	V	8.55	8.03	2.65	5.07	12.09
	L	6.05	6.57	1.93	2.32	8.93
Series 3	H	9.72	9.43	3.54	3.30	14.93
	V	8.25	7.66	3.02	4.31	14.61
	L	5.43	4.37	1.89	3.81	8.13
Series 4	H	9.22	8.38	4.34	3.66	16.66
	V	8.09	7.80	4.03	3.58	15.64
	L	6.26	6.47	1.73	3.92	9.36

**Table 6:** Dissipated energy up to w = 3 mm (W_f,3_) from uniaxial tensile test (in kJ/m^2^).

Test Series	Direction	Mean of W_f,3_	Median of W_f,3_	St. Deviation of W_f,3_	Min. of W_f,3_	Max. of W_f,3_
Series 1	H	6.67	7.02	1.90	3.50	9.51
	V	10.96	10.13	1.71	10.03	14.38
	L	10.11	10.16	0.58	9.37	10.80
Series 2	H	15.07	15.68	5.00	6.34	22.73
	V	10.82	10.13	3.28	6.37	15.34
	L	7.51	7.97	2.49	2.79	11.40
Series 3	H	12.44	11.69	4.71	4.28	19.67
	V	10.77	10.16	4.03	5.45	19.61
	L	6.79	5.25	0.00	8.13	8.13
Series 4	H	11.44	10.89	4.97	4.95	20.08
	V	10.40	10.19	5.30	4.59	20.39
	L	8.16	8.50	2.45	5.08	12.45

**Table 7:** Compressive strength values (in N/mm^2^).

Fiber Content	Number of Specimens	Mean of f_c_	Median of f_c_	St. Deviation of f_c_	Min. of f_c_	Max. of f_c_
0 vol%	12	154.3	154.0	3.44	148.1	160.4
1 vol%	10	167.8	167.1	5.24	160.5	176.8
2 vol%	6	172.5	172.4	6.77	163.0	182.8

**Table 8:** Compressive strength values of the additional series (in N/mm^2^).

Fiber Content	Number of Specimens	Mean of f_c_	Median of f_c_	St. Deviation of f_c_	Min. of f_c_	Max. of f_c_
1 vol%	5	187.1	185.6	4.75	182.0	194.2
2 vol%	5	189.1	190.9	6.31	181.7	197.4

## Data Availability

The data that support the findings of this study are available on request from the corresponding author.
